# Variants of a putative baseplate wedge protein extend the host range of *Pseudomonas* phage K8

**DOI:** 10.1186/s40168-022-01459-w

**Published:** 2023-01-31

**Authors:** Li Sun, Jiajia You, Donghang Li, Zhiqiang Zhang, Xuying Qin, Wenjing Pang, Peize Li, Qingzhu Han, Yueying Li, Zhiwei Huang, Xixi Zhang, Mengxin Gong, Hongjiang Yang

**Affiliations:** Key Laboratory of Industrial Microbiology of the Ministry of Education, Tianjin Key Laboratory of Industrial Microbiology, College of Biotechnology, Tianjin University of Science and Technology, Tianjin, 300457 China

**Keywords:** Phage mutants, Protein variants, Wedge protein, Viral gene reservoir

## Abstract

**Background:**

Narrow host range is a major limitation for phage applications, but phages can evolve expanded host range through adaptations in the receptor-binding proteins.

**Results:**

Here, we report that *Pseudomonas* phage K8 can evolve broader host range and higher killing efficiency at the cost of virion stability. Phage K8 host range mutant K8-T239A carries a mutant version of the putative baseplate wedge protein GP075, termed GP075m. While phage K8 adsorbs to hosts via the O-specific antigen of bacterial LPS, phage K8-T239A uses GP075m to also bind the bacterial core oligosaccharide, enabling infection of bacterial strains resistant to K8 infection due to modified O-specific antigens. This mutation in GP075 also alters inter-protein interactions among phage proteins, and reduces the stability of phage particles to environmental stressors like heat, acidity, and alkalinity. We find that a variety of mutations in *gp075* are widespread in K8 populations, and that the *gp075*-like genes are widely distributed among the domains of life.

**Conclusion:**

Our data show that a typical life history tradeoff occurs between the stability and the host range in the evolution of phage K8. Reservoirs of viral gene variants may be widely present in phage communities, allowing phages to rapidly adapt to any emerging environmental stressors.

Video Abstract

**Supplementary Information:**

The online version contains supplementary material available at 10.1186/s40168-022-01459-w.

## Background

Phages are a group of viruses that hunt bacteria in ecological niches. Adsorption is the first key step for initiation of phage infection and is achieved by specific interactions between viral structural proteins and receptor molecules on the host cell surface. Viral proteins responsible for binding receptors are mainly the components of phage tail tips, including tail fiber proteins, tail spike proteins, tail baseplate proteins, and distal tail proteins. In some cases, phage head filaments are involved in the initial adsorption of phages to host cells. Receptors include various cell-surface structures, which comprise various types of chemical moieties, such as lipopolysaccharides (LPS), outer membrane proteins, flagella, pili, peptidoglycan, teichoic acids, and other cell appendages [1–5].

Just like the emergence of antibiotic resistance, phage-resistant mutants with impaired or altered phage receptors are frequently isolated from various phage-based applications. Fortunately, phages can solve this problem all by themselves through evolution. After bacteria evolve malfunctional receptors that confer resistance to phage infection, adaptations in the phage receptor-binding proteins can enable phages to regain the infectivity of the host cells [6, 7]. One of the well-studied cases is the antagonistic coevolution between *Escherichia coli* cells and the λ phage. Normally phage λ infects the sensitive host cells through the interaction between the viral structural protein J, which is positioned in the tail tip complex, and the outer membrane protein LamB. At low frequencies, a small percentage of the λ phage population can infect the resistant cells which lacks the protein LamB. The new adsorption process is achieved via an alternative interaction between the mutated J protein and another outer membrane protein OmpF. The enormous number of the λ phage population allows the J protein to evolve adaptively for the λ phage to gain the entry into the resistant host cells [8].

Phage K8 is a myovirus that specifically infects the wild-type strain PAK of *Pseudomonas aeruginosa* [9]. This virus encodes a tail fiber protein GP076 acting as a receptor-binding protein binding to O-specific antigen (OSA) LPS for infection. OSA defective strains are often recovered from the K8 lysates, exhibiting the resistance to phage K8 [10, 11].

In this study, we found that the phage K8 population encompassed a small percentage of mutants with an expanded host range, lysing both the wild-type strain PAK and the phage resistant mutants with truncated OSA. Genome sequencing of one mutant phage revealed that no sequence change was identified in the putative tail fiber proteins GP076 and GP078 and other viral structural proteins except that an amino acid substitution T239A was identified in the putative baseplate wedge protein GP075. The molecular basis of the expanded host range carried by the K8 mutant was investigated in this work.

## Results

### Screening of the phage mutants lysing OSA-loss cells

**Fig. 1:**
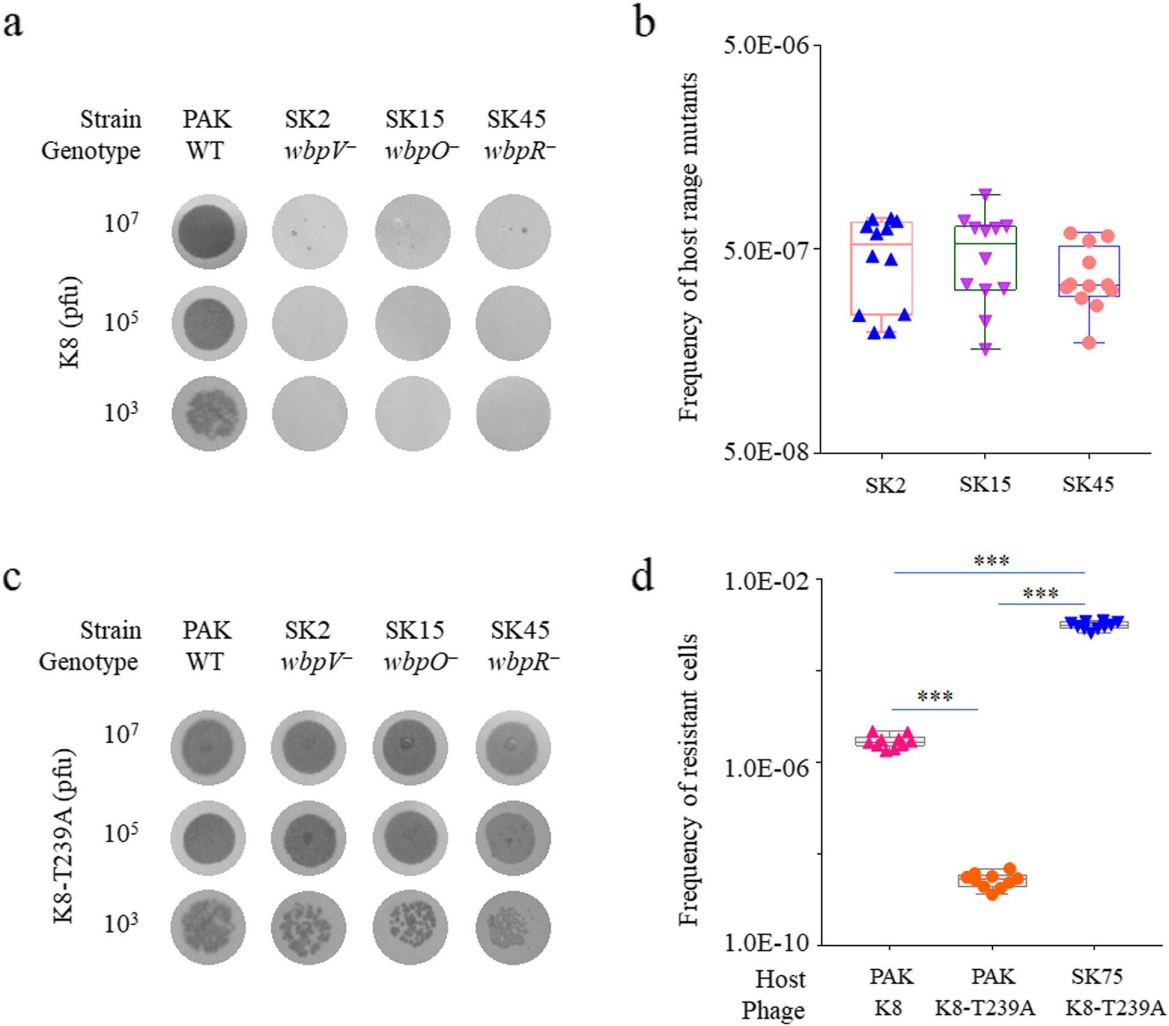
Screening of phage mutants infecting OSA-loss cells. **a** Host range analysis of phage K8 by spotting assay. Indicated amounts of phage K8 were spotted on the bacterial lawns of the indicated strains. Light grey zones represent bacterial lawns. Dark grey zones or dots represent transparent zones or plaques formed in phage infections. **b** Frequency determination of the K8 mutants, which can lyse the OSA-defective strains SK2, SK15, and SK45, in the K8 population. The experiments were independently repeated 12 times. **c** Host range analysis of the mutant phage K8-T239A by spotting assay. Indicated amounts of the mutant phage K8-T239A were spotted on the bacterial lawns of the indicated strains. Light grey zones represent bacterial lawns. Dark grey zones or dots represent transparent zones or plaques formed in phage infections. **d** Frequency determination of the resistant cells. The spontaneous mutation frequencies of the wild-type strain PAK and the OSA-defective strain SK75 were measured when treated with phage K8 and K8-T239A, respectively. The experiments were independently repeated 10 times. One-way ANOVA was performed followed by a Tamhane T2 test to compare the means of the three data groups. ***P < 0.001

*P. aeruginosa* phage K8 binds to OSA LPS for infection, and the OSA-defective mutants of PAK are resistant to phage K8 infection [10]. However, when applied with enormous amounts of phage K8, clear plaques were observed on the lawns of the OSA-defective strains, possibly due to the emergence of mutated phages (Fig. 1a). To estimate the phage mutant frequency in the K8 population, the OSA-defective strains SK2, SK15, and SK45 were used as indicators. Similar results were obtained in the host strains, 4.8 ± 2.1 × 10^−7^, 5.0 ± 2.3 × 10^−7^, and 3.8 ± 1.3 × 10^−7^, respectively (Fig. 1b). One clear plaque was randomly selected from the SK45 lawn and purified after three passages by the double-layer plating method. The purified phage was named K8-T239A and formed circular transparent zones on the lawns of the wild-type strain PAK and the OSA defective strains, SK2, SK15, and SK45, displaying an expanded host range (Fig. 1c). Additionally, the frequency of the spontaneous resistant mutants to phage K8 and K8-T239A was measured in the indicated hosts including SK75 which is also an OSA defective strain, about 3.1 ± 1.0 × 10^−6^ of PAK cells resistant to phage K8, 9.9 ± 1.9 × 10^−4^ of SK75 cells resistant to phage K8-T239A, and only 2.9 ± 1.0 × 10^−9^ of PAK cells resistant to phage K8-T239A. Compared to the K8 group, much less PAK cells were survived in the K8-T239A treatment, indicating that phage K8-T239A may has a relatively high killing efficiency in possible phage application (Fig. 1d).

### Genome sequencing of K8-T239A

To investigate what genetic mutations underlie these phenotypic changes in K8-T239A, we sequenced the K8-T239A genome and compared with the reference genome of phage K8 (accession no. KT736033). Surprisingly, no mutations were observed in the putative tail fiber protein genes *gp076* and *gp078*, the baseplate related protein gene *gp074*, nor the major capsid protein gene *gp057*. Instead, a single base substitution 715G>A was identified in the gene *gp075*, resulting in one amino acid substitution T239A of the mutated version of GP075, GP075m.

### Phage K8-T239A targets core oligosaccharide

**Fig. 2:**
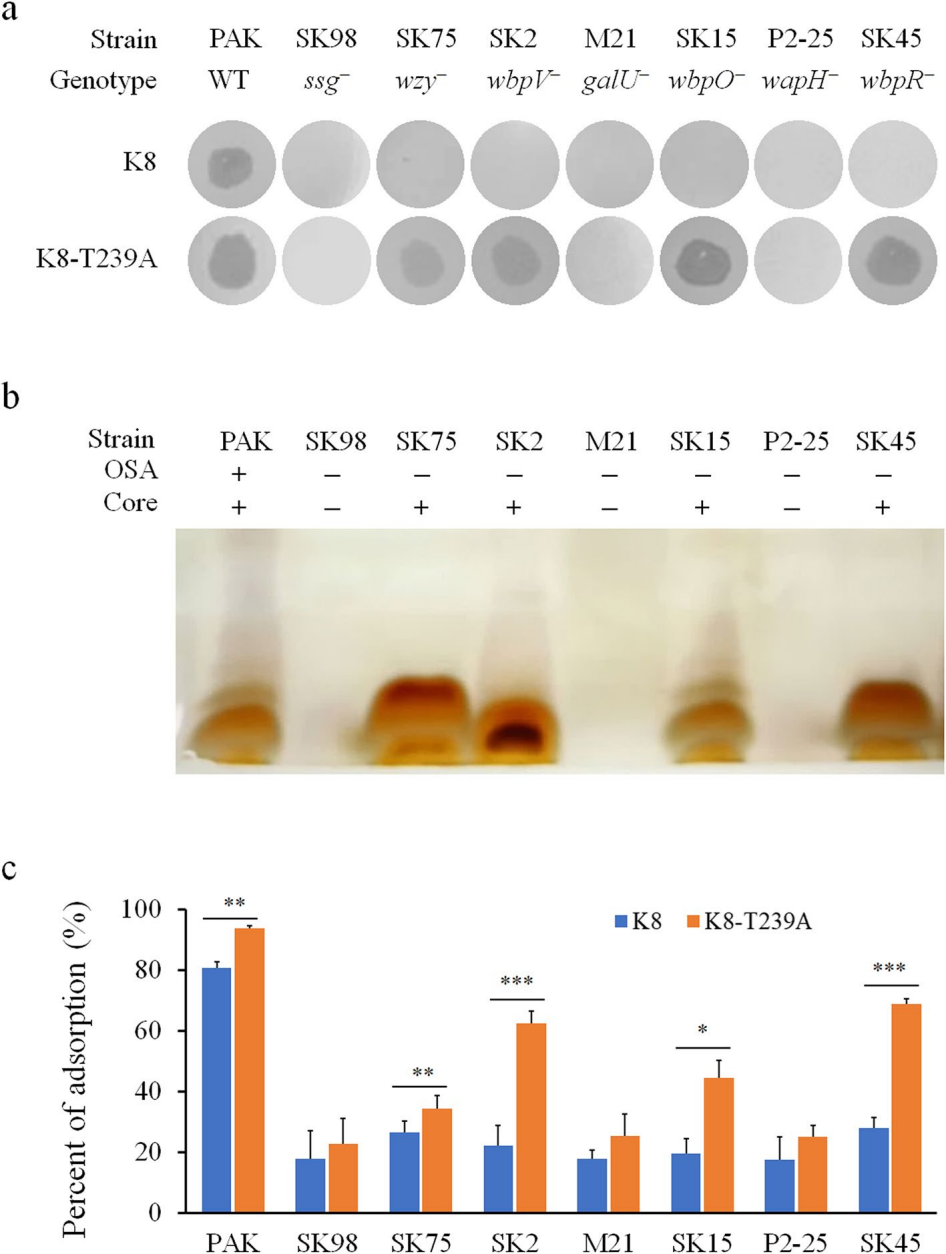
Receptor analysis of phage K8-T239A. **a** Host range analyses of phage K8 and K8-T239A by spotting assay. About 10^6^ phage particles were used in each spot. **b** Tris-Tricine SDS-PAGE of the core oligosaccharide of the indicated strains. OSA: O-specific antigen of LPS. Core: core oligosaccharide. **c** Adsorption analysis of phage K8 and K8-T239A. The adsorption time was 10 min for each test. The experiments were independently repeated three times. One-way ANOVA was used to analyze the difference between the means of the indicated groups treated with K8 or K8-T239A, respectively. *P < 0.05. **P < 0.01. ***P < 0.001

Phage K8-T239A was shown having a broader host range. To investigate the molecular basis of the K8-T239A adsorption, seven PAK derivative strains were used as the indicators in the spotting assay [10]. SK98, M21, and P2-25, with the transposon Tn*5G* inserted in the gene *ssg*, *galU*, and *wapH*, respectively, are deficient in core oligosaccharide and resistant to phage K8-T239A; while the other four strains SK75, SK2, SK15, and SK45, with the Tn*5G* transposon inserted in the OSA synthesis genes, retain core oligosaccharide and are sensitive to phage K8-T239A (Fig. 2a, b), implying that phage K8-T239A possibly recognizes core oligosaccharide as an adsorption receptor. Moreover, K8-T239A displayed higher adsorption percentages than K8 in the sensitive strains including the wild-type strain PAK (Fig. 2c). The data indicated that K8-T239A may simultaneously employ two phage receptors: OSA and core oligosaccharide when adsorbing the PAK cells.

### Reversible adsorption of K8-T239A to core oligosaccharide

**Fig. 3:**
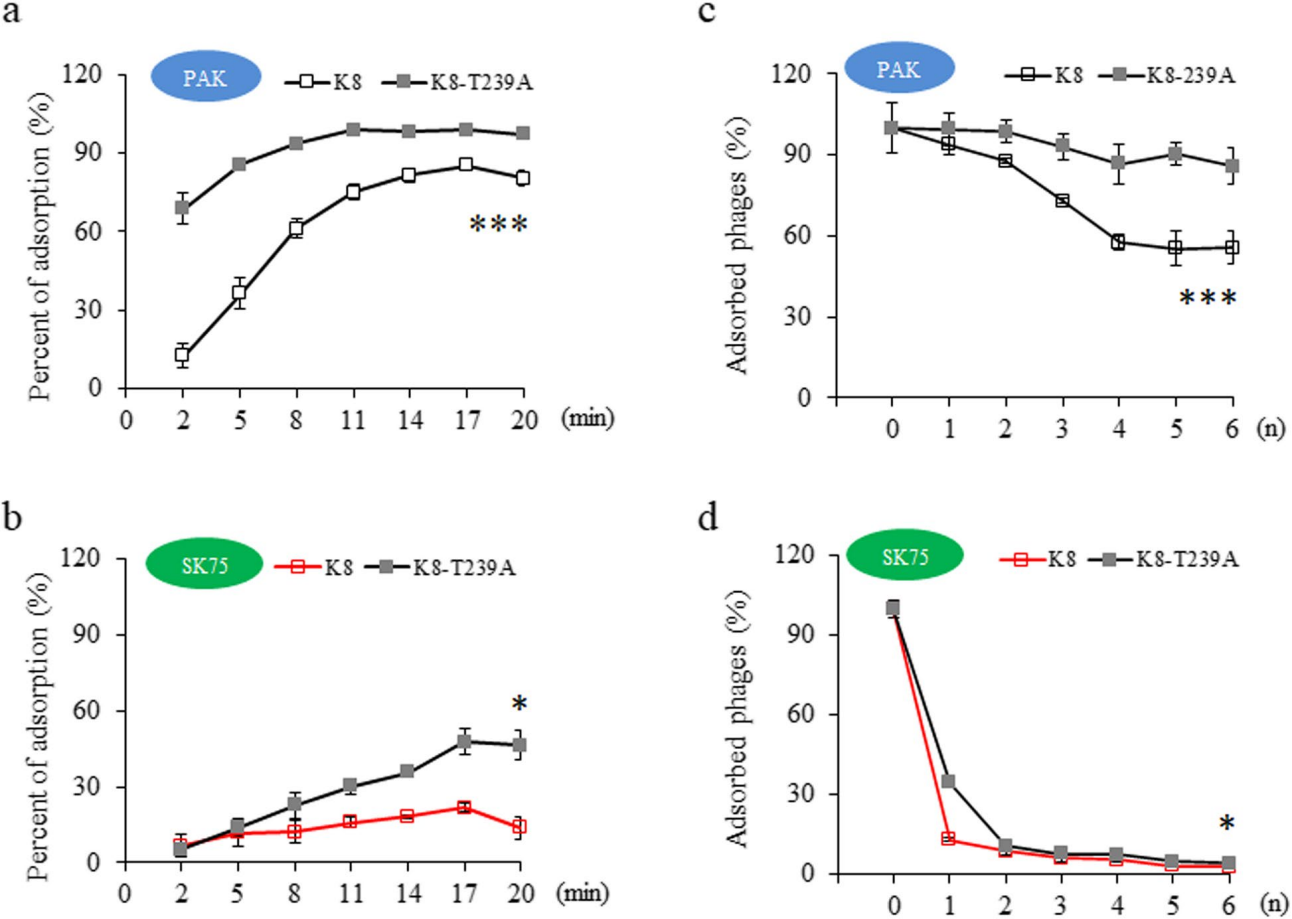
Affinity evaluation between K8-T239A and core oligosaccharide. **a** Time course of adsorption percentage of the indicated phages to the wild-type strain PAK cells. Samples were taken at 3-min intervals. **b** Time course of adsorption percentage of the indicated phages to the OSA-defective strain SK75 cells. The SK75 cells were not sensitive to phage K8 (red line). Samples were taken at 3-min intervals. **c** Resistance of the absorbed phages to washing treatments. The PAK cells were used as indicator. The *x*-axis represented washing times (*n*) of the phage-cell complexes. **d** Resistance of the absorbed phages to washing treatments. The SK75 cells were used as indicator. The *x*-axis represented washing times (*n*) of the phage-cell complexes. The SK75 cells were not sensitive to phage K8 (red line). The experiments were independently replicated three times. A paired samples *t* test was performed to compare the means of the adsorption percentages of the groups. *P < 0.05. **P < 0.01. ***P < 0.001

PAK cells adsorbed significantly more of the K8-T239A virions than K8, while the OSA-defective strain SK75 adsorbed much less of the phage particles than the strain PAK (Fig. 3a, b), implying that the receptor OSA plays a major role in the adsorption process. To observe the strength of adsorption of phage particles to different cell receptors, phages and bacteria were mixed then washed repeatedly, measuring the remaining attached phages after each wash. After washing 4 times, about 86.8 ± 7.3% of K8-T239A and 57.6 ± 3.0% of K8 were retained on the surface of the PAK cells without further significant reductions (Fig. 3c). The data implied that the interaction between OSA and phage K8 or K8-T239A was the primary association. Only 34.3 ± 8.3% of K8-T239A and 12.9 ± 3.6% of K8 were retained on the SK75 cell surface after the first round of washing, and both phages were easily removed from the SK75 cell surface in the following washing steps (Fig. 3d). The data implied that the association between the receptor core oligosaccharide and phage K8-T239A was weak and reversible. Reversible adsorptions are often observed in phage-bacteria interactions, e.g., phage SPP1 initiates adsorption by rapidly attaching to the reversible receptor, wall teichoic acid (WTA), and then form an irreversible association with the membrane protein YueB [12].

### GP075m directly binds to core oligosaccharide

**Fig. 4:**
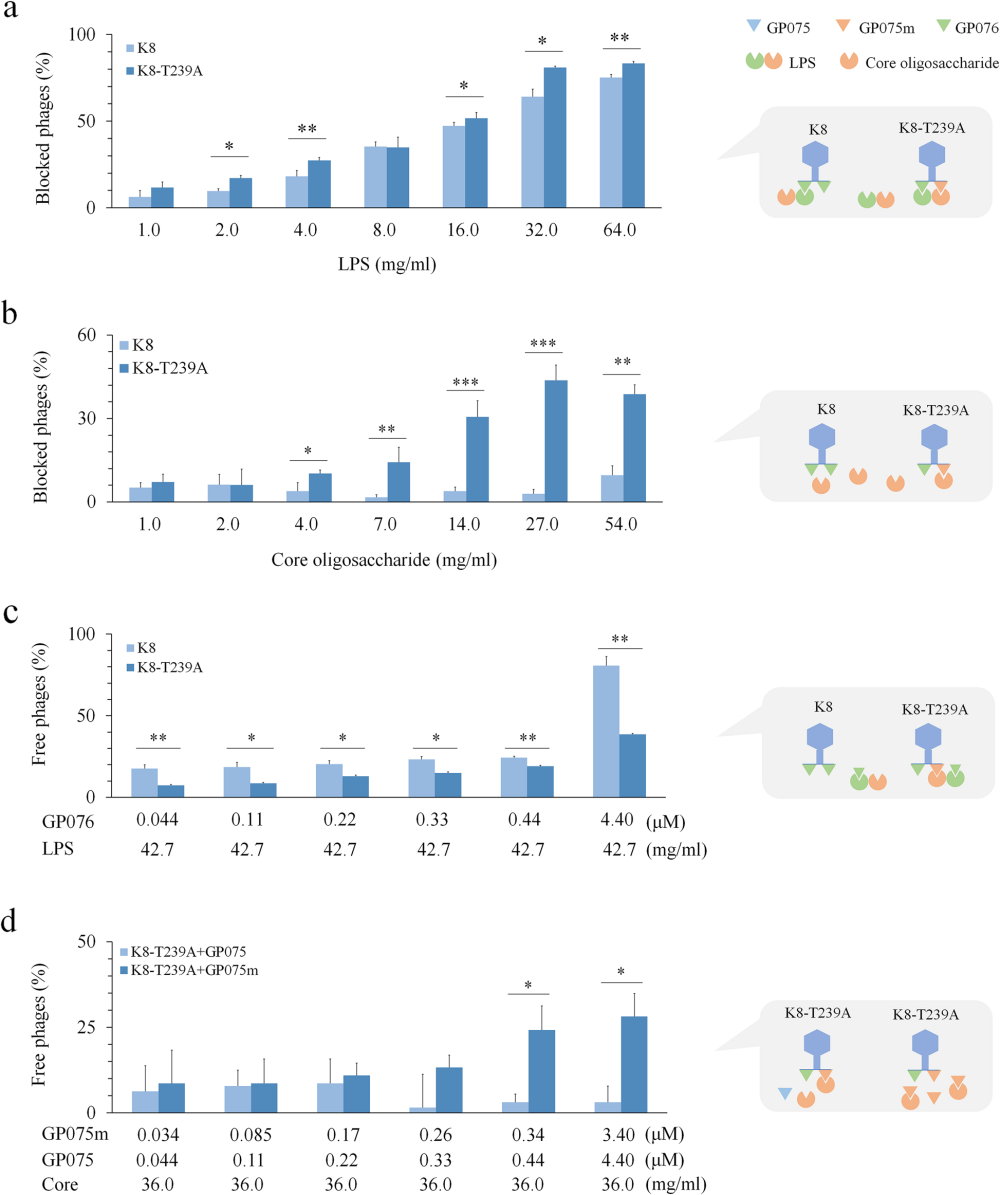
Identification of the association between GP075m and core oligosaccharide. **a** Blocking efficiency of LPS to the indicated phages (left panel). Schematic diagram of the associations between the indicated phages and LPS (right panel). **b** Blocking efficiency of core oligosaccharide to the indicated phages (left panel). Schematic diagram of the associations between the indicated phages and core oligosaccharide (right panel). **c** Binding efficiency of the tail fiber protein GP076 to LPS (left panel). Schematic diagram of the associations between the protein GP076 and LPS (right panel). **d** Binding efficiency of the protein GP075m to core oligosaccharide (left panel). The protein GP075 was used as the control. Schematic diagram of the associations between GP075m and core oligosaccharide (right panel). PAK was used as the indicator strain for measurement of the phage titers in all experiments. The experiments were independently repeated three times. One-way ANOVA was used to analyze the mean difference of the indicated different treatments. *P < 0.05. **P < 0.01. ***P < 0.001

To determine phage binding to OSA and the core oligosaccharide components of LPS, LPS from cells with or without OSA was extracted. Then, the phage suspensions were exposed to this purified LPS across a range of LPS concentrations. In the blocking analyses, the more LPS or core oligosaccharide were applied, the more phages were blocked; phage K8 was not inhibited by core oligosaccharide; more K8-T239A were blocked than K8 in the presence of the same amount of LPS or core oligosaccharide (Fig. 4a, b), suggesting that GP075m encoded by phage K8-T239A likely uses core oligosaccharide as a binding target. The binding efficiency of GP075m to OSA or core oligosaccharide was measured with phage KA and K8-T239A, respectively. The results showed that GP075m neutralized core oligosaccharide and GP076 neutralized LPS, while no binding was detected between GP075 and core oligosaccharide (Fig. 4c, d). These data provided evidence that the phage tail protein GP076 and GP075m directly bind to the two phage receptors, OSA, and core oligosaccharide, respectively.

### GP075 and its variant GP075m are structural proteins

Genetic and biochemical data supported that the protein variant GP075m served as an additional receptor-binding protein of phage K8-T239A, and it was presumed that GP075m is a structural component of the virions. Subsequently, the particles of phage K8 and K8-T239A were harvested, purified, and subjected to the examination by mass spectrometry, respectively. Both the hypothetical protein GP075 and its variant GP075m were identified in the K8 and K8-T239A particles, respectively (Supplementary Dataset 1). The results implied that the hypothetical protein GP075 and its variant GP075m were the structural components of phage K8 and K8-T239A, respectively.

### Structure prediction of GP075 and its variants

With the online software BLASTP, either GP075 or its variant GP075m was predicted as a hypothetical phage protein containing a conserved protein domain of unknown function (DUF2612). Protein structure homology was further analyzed by using the software HHpred. The predicted results showed that all the analyzed proteins, GP075, GP075m, and GP075-D7 which has one 7-aa identical duplication (APWYSVG) between the 113th and 114th amino acid residual of GP075 identified in the mutant phage K8-D7 (Supplementary Table S3), were structurally homologous to the baseplate wedge protein Gp6 of *E. coli* phage T4 with high probabilities greater than 95%, suggesting that these proteins may share similar protein structure, likely being part of the baseplate complex [13].

The structure difference of GP075, GP075m, and GP075-D7 was further predicted by using the online tool SWISS-MODEL (https://swissmodel.expasy.org/). Compared with GP075, the alpha helix content was lower in GP075m and GP075-D7, and the beta strand content was higher in GP075-D7. The data indicated that the amino acid substitution or the duplication brings the changes of the secondary structure, which may be responsible for the binding capacity of the GP075 variants to core oligosaccharide (Supplementary Figure S1). Our data are consistent with the previous work that amino acid substitutions may affect protein structures, functions, and the related phenotypes [14].

### In vitro aggregation of GP075 and its variant GP075m

Previous studies showed that phage structural proteins tend to aggregate in polymers in solution, and it might be related with the in vivo oligomeric state of the phage structural proteins [15]. We expressed and purified the putative structural proteins for the in vitro characterizations (Supplementary Figure S2). Preliminary data of the Native-PAGE revealed that both GP075 and GP075m were likely aggregated in solutions. Addition of the detergent chemical, sodium dodecyl sulfate (SDS), disaggregated the polymers of GP075 and GP075m in the sample solutions (Supplementary Figure S3). Aggregation degree was quantitively estimated by the SEC-MALS analysis with bovine serum albumin (BSA) as the control [16]. The self-polymerization degree was 1.0, 81.2, and 185.3 for the proteins BSA, GP075, and GP075m, respectively (Supplementary Table S3). This is consistent with past work showing that phage structural proteins can form oligomers of varying aggregation degrees in solution [15]. In addition, GP075 and GP075m had different polymerization degrees, which may be explained by the previous work that amino acid substitutions introduced on the protein surface/interface may cause association or dissociation of homooligomers [17]. Although both GP075 and GP075m aggregate in oligomers, their state in the virions remained to be unveiled.

### Possible interactions of the phage proteins

To further investigate the possible role of the GP075 proteins, the BacterioMatch two-hybrid system was used to screen possible interactions between the structural proteins encoded by phage K8, K8-T239A, and K8-D7 which encodes the variant protein GP075-D7 (Supplementary Table S3), respectively. Growth rates of the reporter strains carrying the structure protein genes were measured, including two putative baseplate protein genes *gp072* and *gp074*, two putative tail fiber protein genes *gp076* and *gp078*, and the putative baseplate wedge protein genes *gp075*, *gp075m*, and *gp075-D7*. The screening assay revealed that GP074 had probable associations with GP075, GP075m, GP075-D7, and GP076, respectively, whereas GP076 may only interact with GP075 (Supplementary Table S4). The interaction data suggested that GP075 and its variants were possibly as a part of the baseplate complex.

### Stability of the K8 phages

Thermostability was analyzed among phage K8 and its derivatives carrying the GP075m-like proteins. When treated at 30 °C or 40 °C, all phage mutants displayed similar heat tolerance to K8 except K8-D7. When treated at higher temperatures, phage K8 showed a stronger heat tolerance characteristic compared with its derivatives, suggesting that phage K8 may have a robust virion structure (Supplementary Figure S4).

**Fig. 5:**
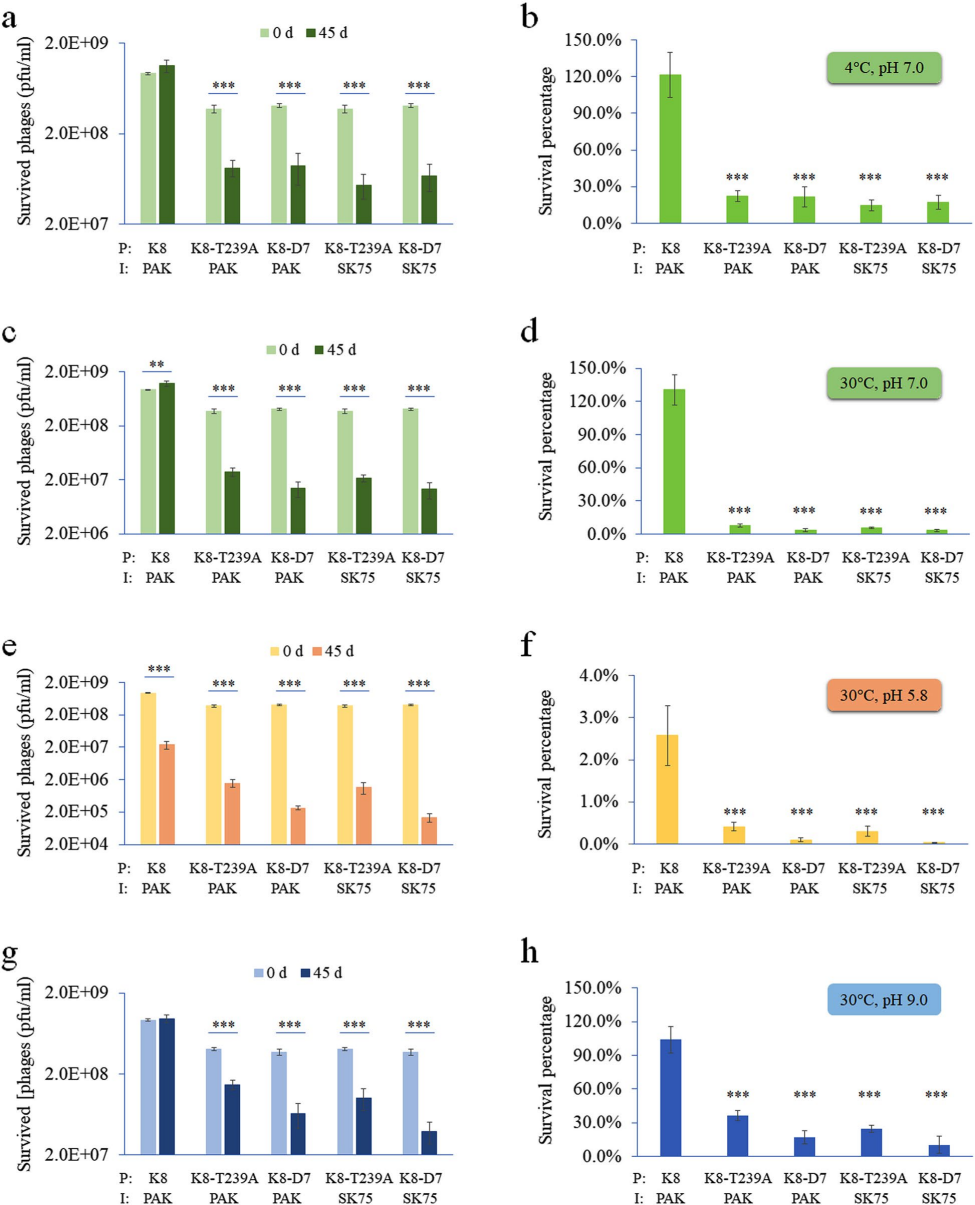
Stability analysis of phage K8 and its derivatives. Phage K8, K8-T239A, and K8-D7 were diluted in the indicated buffers and kept at different temperatures for a period of 45 days. **a**, **b**The indicated phages were diluted in 0.01 mol/l PBS (phosphate buffered saline, pH 7.0) and kept at 4 °C for 45 days. **c**, **d** The indicated phages were diluted in 0.01 mol/l PBS (pH 7.0) and kept at 30 °C for 45 days. e and f: The indicated phages were diluted in 0.01 mol/l Tris-HCl buffer (pH 5.8) and kept at 30 °C for 45 days. **g**, **h** The indicated phages were diluted in 0.01 mol/l PBS (pH 9.0) and kept at 30 °C for 45 days. Survived phage titers were measured (left panels) and the related survival percentages were calculated for each phage (right panels). The experiments were independently repeated three times. P: the tested phage. I: bacterial strain used as indicator. One-way ANOVA was used to compare the means of the phage titers tested at day 0 and day 45, respectively (left panels). Differences among the mean survival percentages of the groups were analyzed with one-way ANOVA followed by a Tamhane T2 test (right panels). *P < 0.05. **P < 0.01. ***P < 0.001

Long-term stability of the K8 phages was estimated under the conditions with different ambient temperatures and acidities. After storage for 45 days, phage K8 was quite stable under all the conditions except the acidic stressor, in which the infectious virions significantly decreased during the incubation process. Similar reductions were observed in all the samples of the K8 derivatives under all conditions even in the neutral solution at 4 °C. The data suggested that phage K8 is likely the most robust biosystem in the tested phages (Fig. 5a, c, e, and g). Interestingly the titers of phage K8 showed slightly increased after the storage period of 45 days in pH 7.0 and pH 9.0 conditions (Fig. 5a, c, and g). We assumed that the phage lysates contained undetermined amounts of free phage receptors, OSA and core oligosaccharide, from the lysis process of the host cells by the phages, and a certain amount of the phage particles were blocked in the lysates. In the survival tests, the blocked phages were released from the phage-LPS complexes due to the 300-fold dilutions, resulting in some increase of the indicated phages. For more direct comparisons, survival percentages were calculated used for comparisons. In all conditions, the survival percentages of the mutants K8-T239A and K8-D7 were significantly lower than that of phage K8 (Fig. 5b, d, f, and h). Taken together, the data indicated that phage K8 showed a great tolerance to diverse conditions and it may be the reason why phage K8 not its derivatives were isolated from the environmental sample. The structural differences possibly have the long-term effects on the virion stability in diverse ecological systems.

Two strains were used in the analysis as the indicator, PAK and SK75. The former strain can form two links with the mutant phages K8-T239A and K8-D7, while the latter one can only form one link with the mutant phages. The results showed that more phages were detected with the indicator strain PAK than that with SK75 (Fig. 5b, d, f, and h). The data further indicated that GP075m or GP075-D7 are likely complementing the adsorption activity of the phage tail fiber protein GP076 by binding to another phage receptor core oligosaccharide.

### Diversity of the gp075 gene

Given the existence of the K8 subpopulations which have the broader host range driven by variation in *gp075*, it is likely that other variants of *gp075* with ramifications for host range will exist. We initially isolated 41 strains of the K8 mutants, and they can lyse both the wild-type strain PAK and the OSA defective strains, having the same host range as K8-T239A, and hence they were referred as “the K8-T239A-like mutants”. Sequence analysis of the *gp075* gene revealed that among the K8-T239-like mutants, 5 mutants have a 21-bp identical duplication (5′ GCTCCATGGTACTCGGTCGGT 3′) between the 339th and 340th nucleotides or a 7-aa identical duplication (APWYSV) between the 113th and 114th amino acid residues (named D7), 25 mutants have diverse single amino acid substitutions, and the remaining 11 mutants have no mutations in GP075 (Supplementary Table S5).

**Fig. 6:**
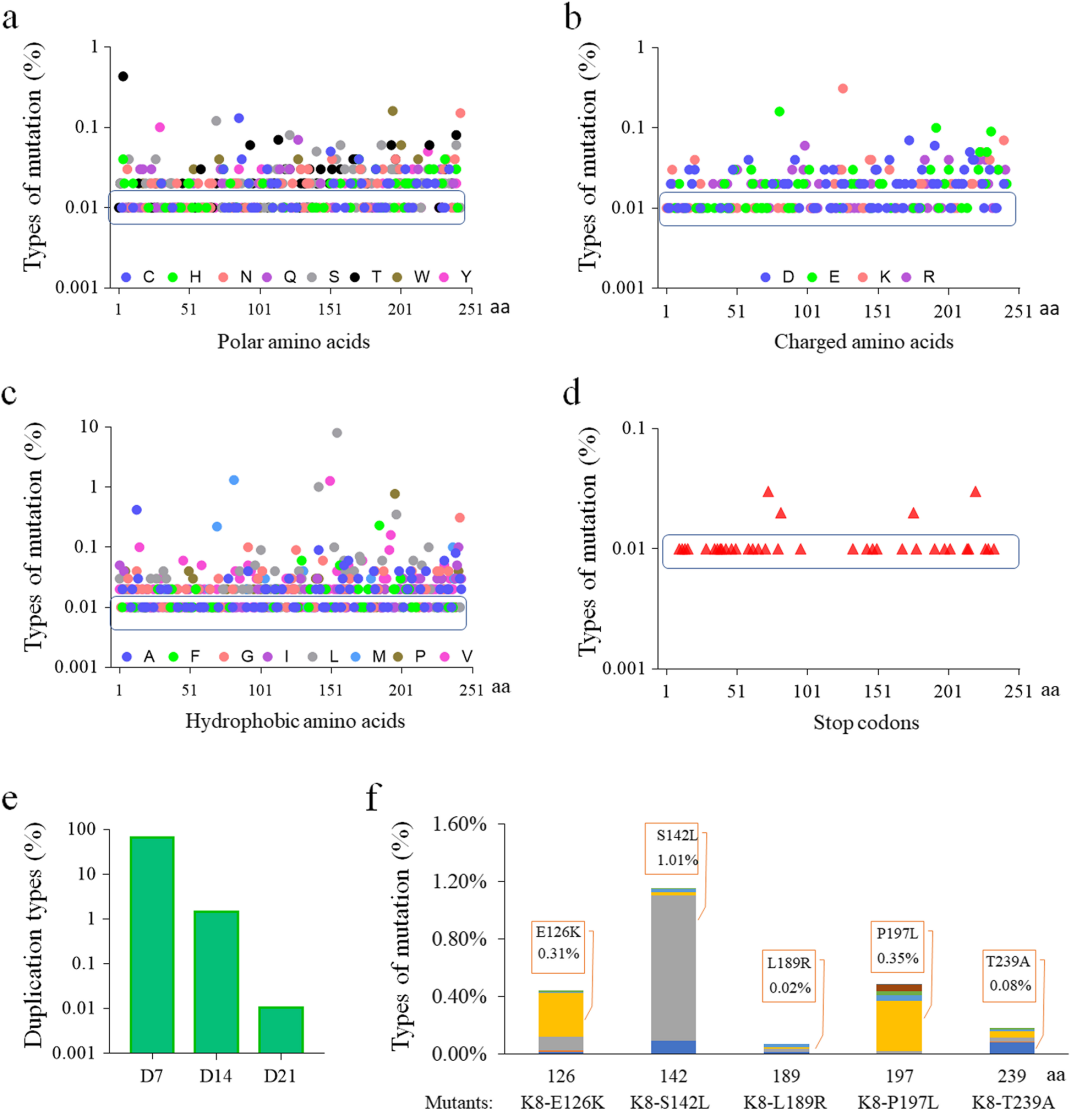
Amplicon sequencing of the genetic diversity of the GP075 variant genes. GP075 was composed of 243 amino acid residues and their sequence positions were labeled by the natural numbers on the *x*-axes. The single letters (not including that of Fig. 6e) represented the amino acids. **a** Percent of the polar amino acid substitutions in the GP075 variants. **b** Percent of the charged amino acid substitutions in the GP075 variants. **c** Percent of the hydrophobic amino acid substitutions in the GP075 variants. **d** Percent of the stop codons in the GP075 variants. e. Percent of the duplications in the GP075 variants, D7, D14, and D21. Duplications were located between the 113th and 114th amino acid residues of GP075, D7 with one additional copy of the 7-aa peptide (APWYSV), D14 with 2 additional contiguous copies of the 7-aa peptide, and D21 with 3 additional contiguous copies of the 7-aa peptide. **f** Percent of the amino acid substitutions of GP075 identified in the isolated K8-T239A-like mutants. The positions of the amino acid substitutions of GP075 were labeled on the *x*-axis. Color bars represented the percent of the amino acid substitutions at the specific sites of the GP075 variants revealed by amplicon sequencing of the pool of 2000 phage plaques collected from the bacterial lawns of the OSA-defective strains. Mutations with the percentage value less than 0.02% (dots within the blue rectangles) were considered as experimental noise generated from the analysis. Mutants: the K8-T239A-like mutants isolated from the single phage plaques formed on the lawns of the OSA defective strains

To fully understand the genetic diversity of the *gp075* gene in the group of the K8-T239A-like mutants, a total of 2000 phage plaques were collected and pooled together for the extraction of the viral metagenomic DNA, that was used as the template for amplicon sequencing to investigate the *gp075* gene alleles in the collected sample (Supplementary Dataset 2). A great diversity of amino acid substitutions was identified, including polar amino acids, charged amino acids, and hydrophobic amino acids. Four dominant substitutions were found in the substitutions, including I82M (1.3%), S142L (1.0%), A150V (1.3%), and F155L (8.0%) (Fig. 6a–c). Duplications were identified in 66.6% of the amplicons, including D7 (65.1%) with one additional copy of the 7-aa, D14 (1.5%) with two additional continuous copies of the 7-aa, and D21 (0.01%) with three additional continuous copies of the 7-aa (Fig. 6e). The results indicated that the allele pool of the gene *gp075* is highly divergent in the K8-T239A-like mutants, and the *gp075-D7* is the primary mutation event.

To verify the amplicon sequencing data, we examined if the data included the GP075 variants identified in the isolated K8-T239A-like mutants (Supplementary Table S5). The results showed that the GP075 variants of K8-E126K, K8-S142L, K8-L189R, K8-P197L, and K8-T239A were also identified in the amplicon sequencing data with the percentages of 0.31%, 1.01%. 0.02%. 0.35%, and 0.08%, respectively, and they were also the most frequent mutation events in the specific sites (Fig. 6f). Our data indicated that mutations with the percentages of 0.02% or higher might be considered as true mutations, not errors or noises generated from the sequencing analysis. On the basis of the established cutoff value, mutations with the percentage less than 0.02% would be treated as experimental noise (Fig. 6a–c). For stop codon mutations, only four sites with the percentage a bit greater (0.03%) than or equal to the cutoff value (Fig. 6d), suggesting that some mutant phages might encode the inactivated versions of the GP075 protein.

### Repeats of the K8 genome

Duplicate sequences have been identified in a few phage proteins, e.g., duplication of an 11-aa sequence unit in the tape measure protein [18], duplication of a short amino acid sequence in the long tail fiber adhesin Gp37 [19], and an allele pool of the phage portal protein gene [20]. In the eukaryotic genomes, repetitive DNA sequences have been considered as the driven force causing gene or genome evolution with segmental duplications [21]. With the software RepeatAround 2.1, we found that the K8 genome was crowded with various sequence repeats. However, the role of the repeats predicted in the K8 genome remains to be unveiled.

### Distribution of the GP075-like proteins

**Fig. 7:**
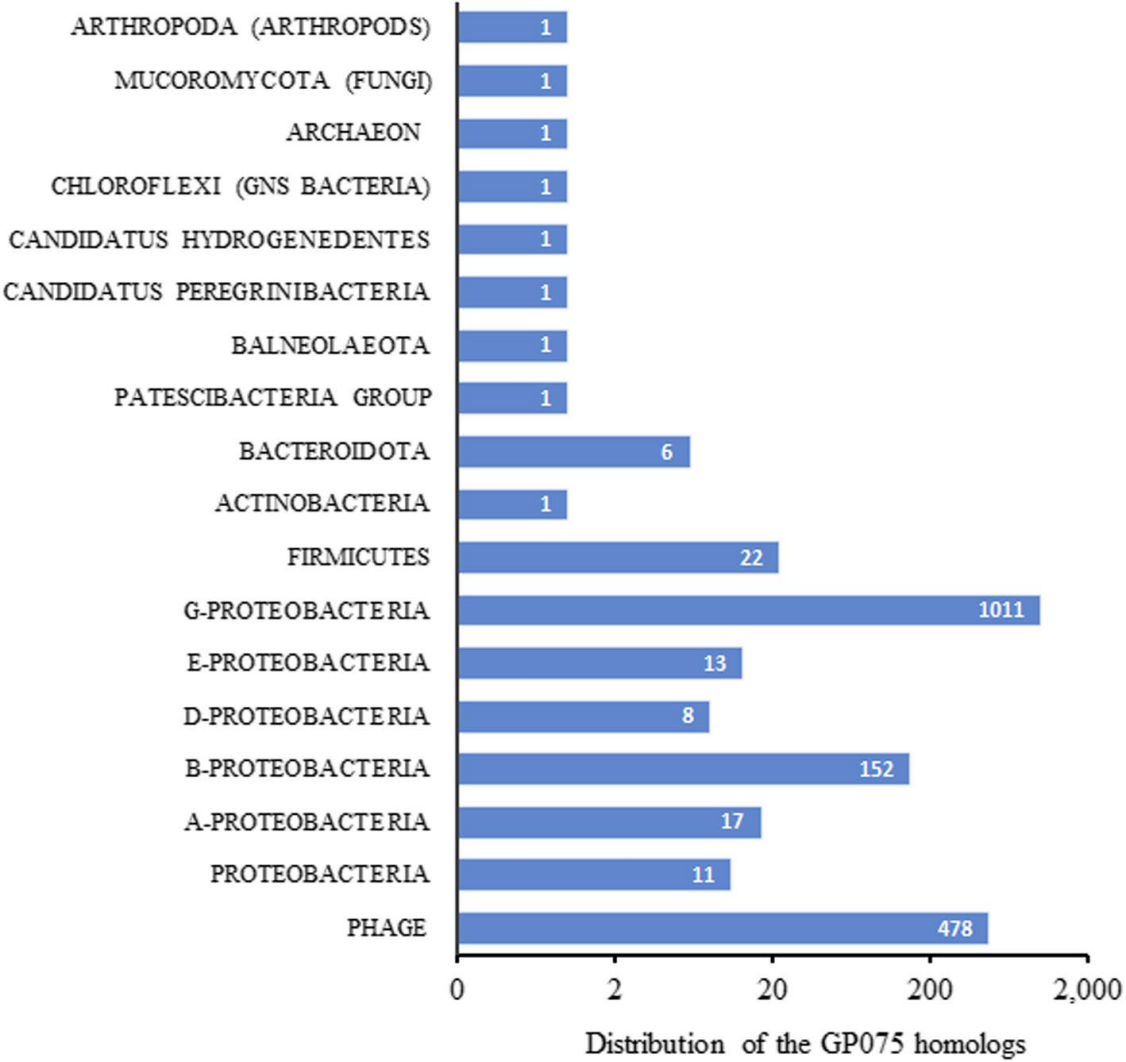
Distribution of the GP075-like proteins. A total of 1727 protein homologs of GP075 were found in various taxonomy taxa of phylum or class with an *e* value cutoff of 0.001. The GP075 homolog numbers of each taxon were labeled in the corresponding bars. The accession numbers of the GP075-like proteins were listed in the Supplementary Dataset 3. A-Proteobacteria: α-Proteobacteria. B-Proteobacteria: β-Proteobacteria. D-Proteobacteria: δ-Proteobacteria. E-Proteobacteria: ε-Proteobacteria. G-Proteobacteria: γ-Proteobacteria

To further illustrate the possible function of GP075, the abundance and diversity of the GP075 homologs were explored. Using the *E* value 0.001 as the cutoff, totally 1727 proteins were identified as the GP075 homologs, mainly including the proteins of phages (478), proteobacteria (11), γ-proteobacteria (1011), β-proteobacteria (152), α-proteobacteria (17), ε-proteobacteria (13), δ-proteobacteria (8), firmicutes (22), and Bacteroidota (6) (Fig. 7 and Supplementary Dataset 3). Although the GP075-like proteins are widely distributed in bacteria, phages, and other taxa, their functions remain unknown in different microorganisms.

### Adaptation of GP075 is not the only way

Among the 41 isolated K8 mutants with the same host range as K8-T39A, no mutation was found in the *gp075* gene of K8-X (Supplementary Table S5). The K8-X genome was sequenced, and was aligned with the reference genome of K8 (accession no. KT736033). The data revealed that phage K8-X has two mutated phage proteins, the hypothetical protein GP011 with one amino acid substitution T42A and the tail fiber protein GP076 with an amino acid substitution D18A. The results suggested that phage K8-X possibly employs another adsorption way, different from phage K8-T239A, for host infection (Supplementary Table S5).

## Discussion

In our work, we found a number of phage mutants with a broader host range within the K8 community when using the non-permissive strains, OSA defective strains of PAK, as the indicators. Most of them have the 7-aa duplications and amino acid substitutions within the coding region of a putative baseplate wedge protein gene *gp075*. The GP075 variants was identified as an additional receptor-binding protein, adhering to core oligosaccharide for phage infection. Our data indicated that phages can overcome phage resistance in natural ecosystem through evolution of putative viral structural proteins.

Phages usually have their host ranges extended through the adaption of receptor-binding proteins in evolution to regain entry to receptor-impaired cells, e.g., *E. coli* phage λ, *Pseudomonas* phage KPP22, *Pseudomonas* phage JG004, and *B. subtilis* phage SPO1 [11, 22–25]. In our work, no amino acids alteration was observed in the two putative phage tail fiber proteins GP076 and GP078 of the K8 derivatives. Instead, GP075m, the variant of the putative baseplate wedge protein GP075, is identified evolved as an extra receptor-binding protein, adsorbing core oligosaccharide other than the known receptor OSA LPS. The association between GP075m and core oligosaccharide can independently dock phage particles on cell surface, leading to a successful infection. Our data imply that the adaptation of tail fiber proteins, e.g., GP076, which usually function as receptor-binding proteins, may not be the only way for phage to overcome the receptor-impaired mutants. Other viral proteins such as the putative baseplate wedge protein may also be involved in phage evolution in responding to environmental changes.

The K8 derivatives evolve a mutation in the GP075 variants that confers binding ability to core oligosaccharide not present with the “wild-type” GP075, arming the K8 mutant phages with two receptor-binding proteins, which are also found in other phages, e.g., *E. coli* phage T4 [1], *Vibrio cholerae* phage VP3 [26, 27], *E. coli* phage ΦK1-5 [28], and *Staphylococcus aureus* phage ΦSA012 [29]. The K8 derivatives can fulfill proliferation process through adsorption via both or either of the two receptors, exhibiting an extended host range. In addition, the killing efficiency of phage K8-T239A is significantly increased in the inhibition of PAK cells, since the OSA-loss or truncated mutants which are frequently recovered from the K8 treatment can be removed by the K8-T239A-like phages, showing great potential for application.

HHpred predicted the protein GP075 and its variants are structurally similar to the protein Gp6 of *E. coli* phage T4 with relatively high probabilities, suggesting that GP075 and GP075m may have similar protein structure to Gp6 [30], though they bear no significant sequence homology. Gp6 is a structural protein required for the assembly of the baseplate wedge structure along with other phage proteins [13], which plays key roles in virion assembly and sheath contraction of phage T4 [31, 32]. However, Gp6 is a monomer in solution, different from GP075 or GP075m. The result suggested GP075 and GP075m may play a different role from the T4 phage protein Gp6.

Tail fiber assembly (Tfa) proteins are a large family of chaperones that are involved in folding and assembly of phage tail fibers. The Tfa protein Tfa_Mu_ of phage Mu is involved in tail fiber folding, associated with the distal tip of the tail fiber, and bound to the receptor LPS [33]. In our work, GP075 is proven possibly associated with the tail fiber protein GP076 but not bound to the phage receptor OSA, while GP075m is not linked to the tail fiber protein GP076 and independently recognizes core oligosaccharide, forming the second link between the host cells and phages. More experiments are required to further explore the possible functions of GP075 and GP075m.

Long-term survival percentages of K8 and the derivatives were estimated under the conditions that mimic natural environments and the results showed that the mutant phages are more fragile relative to the wild-type phage K8 under the indicated stressors. The alterations of the amino acids sequence of the viral protein GP075 may not only extend the host ranges, but also introduce the structural instability of the K8 derivatives, indicating that a typical tradeoff occurs between the generation of a novel function of the protein GP075 and the destabilization of the virion integrity. Our data were consistent with the findings that tradeoffs are widely present in life history in phage evolution [34–37].

## Conclusions

GP075m, which is the mutated putative baseplate wedge protein GP075, makes the phage K8 derivatives propagate in the non-permissive cells of phage K8 and kill more PAK cells. The phenotypic alternations emergence of the K8 derivatives are associated with loss of virion structure stability, which is the typical life history tradeoff of phage evolution. Viral gene variants may be abundant in phage communities. Combination of different viral gene alleles may result in the emergence of genetically and phenotypically diverse subpopulations within phage community, allowing phage to evolve novel traits to adapt to environmental changes. Our data may provide insight into the molecular basis of phage evolution in extending host range.

## Materials and methods

### Bacterial strains and growth conditions

*Pseudomonas aeruginosa* PAK and its Tn*5G* insertion mutants were used as indicators in phage sensitivity test. *P. aeruginosa* phages K5, K8, and C11 were used in the analyses. *P. aeruginosa* phage K8-D7, K8-E126K, K8-S142L, K8-L189R, K8-P197L, K8-T239A, and K8-X were the derivatives of phage K8. *Escherichia coli* strain DH5α was used for construction of recombinant plasmids. *E. coli* strain M15 was used for the expression of His-tagged proteins via the vector pQE30. *E*. *coli* strain RS was used in the protein interaction assay. Luria-Bertani (LB) medium (10 g/l peptone, 5 g/l yeast extract, and 10 g/l sodium chloride) supplemented with appropriate antibiotics was used for routine cultivation. All bacterial strains, plasmids, and phages used in this work were included in the Supplementary Table S1. Primers used in this work were listed in the Supplementary Table S2. Unless otherwise specified, all incubations were performed at 37 °C.

### Spotting assay

The indicated bacterial strains were grown to exponential phase at an OD_600_ of 0.6. The double-layer plate was made by mixing 200 μl of the exponential culture of with 3 ml of 0.5% melted agar (50 °C) and poured on top of the 1.5% L-agar layer in petri dish. The dishes were kept at room temperature for 15 min and 1 μl of the tested phage lysate (about 10^8^ pfu/ml) was spot on them. The plates were incubated at 37 °C for 4 h for observation of transparent zones.

### Spontaneous mutation frequency assay

Population structure of the phage K8 community was investigated by host range analysis. In brief, the lysate of phage K8 was prepared using with the strain PAK as host. OSA defective strains SK2, SK15, and SK45 were used as the indicators to determine the frequency of the K8 mutants. For each analysis, about 10^9^ phage particles and 10^8^ bacterial cells at logarithmic phase (OD_600_=0.6) were mixed with 5 ml 0.5% agar and poured on the top of 1.5% L-agar layer in a petri dish. Plaques were recorded after incubation for 6 h. Triplicate samples were tested in each analysis and the experiments were independently repeated 12 times. Multiple comparisons of the three data groups were performed by using one-way ANOVA followed by a Tamhane T2 test.

Frequency of phage-resistant mutants was measured in the cultures treated with the indicated phage lysates. Overnight cultures of the host strains were serially diluted and bacteria numbers were enumerated by plating the 100-μl dilutions on L-agar plates. To test the number of phage-resistant mutants in the bacterial population, equal amounts of phage particles (10^8^ pfu) were mixed with the 1-ml dilutions of the bacterial cultures followed by incubation at 200 rpm for 20 h. The cloudy tubes with the most-diluted cultures should contain 1 to 9 phage-resistant mutant cells and we took the median value of 5 in the calculation of frequencies. The experiments were independently replicated 10 times. Multiple comparisons were performed to analyze the difference between the means of the indicated data groups with one-way ANOVA followed by a Tamhane T2 test.

### Adsorption analysis

Adsorption analysis was performed by mixing 200 μl of exponential cultures of the indicated strains (OD_600_ = 0.6) with 100 μl of phage lysate at a MOI of about 0.001. The mixtures were kept statically at room temperature for 10 min then spun at 10000×*g* for 30 s. The supernatants were collected and serially diluted. Phage titer was determined for calculation of the adsorption percentages. The experiments were independently replicated three times. One-way ANOVA was performed to compare the means of the adsorption percentages of the strains adsorbed by different phages.

### Time course of adsorption percentage

The time course of adsorption percentage was plotted by sampling at 3-min intervals and the titer of free phages in the supernatants was determined for calculation of the percent of adsorption. The experiments were independently replicated three times. A paired samples *t* test was performed to compare the means of the adsorption percentages.

### Reversibility of phage adsorption

To determine if an adsorption process is reversible, the percentage of adsorbed phages on cell surface was evaluated during a washing process. In brief, a 650-μl culture of the indicated strains (OD_600_ = 0.6) was mixed with an equal volume of the phage lysate at a MOI of about 0.001. One hundred microliters of mixture was removed for determination of the total phage titer. The remaining mixture was kept statically for 1 min at room temperature and subjected to centrifugation at 10000×*g* for 30 s. The pellet was resuspended in 1200 μl LB medium. Another 100 μl mixture was removed for determination of phage titers after kept statically at room temperature for 1 min. The process was repeated for five more times. The experiments were independently replicated three times. A paired samples *t* test was performed to compare the means of the percentages of the adsorbed phages.

### Genome sequencing and comparative genomic analysis

As described previously, the indicated phage particles were collected and purified with the precipitation of PEG6000 (10%) [10]. Then the resuspended phages were treated with 50 μg/ml proteinase K and 0.5% SDS at 56 °C for 4 h. After repeat extractions with phenol-chloroform and chloroform, the genomic DNA was precipitated with ethanol. Subsequently the phage genomic DNA was sequenced at the Illumina HiSeq 2500 system (https://www.genewiz.com/). Q scores including Q20 and Q30 were used for assessing the quality of sequencing data. The assembled genome was further curated by aligning with the reference genome of phage K8 (KT736033) with the online software BLASTN of NCBI.

### Overexpression of the phage proteins

Phage genomic DNA was used as template for amplification of the phage protein genes with the indicated primers (Supplementary Table S2). The PCR (polymerase chain reaction) products were cloned into the vector pQE30 to overexpress the his-tagged phage proteins, respectively. *E. coli* cells with the inclusion bodies were suspended in the lysis buffer (50 mmol/l Tris-HCl, 250 mmol/l NaCl, 1 mmol/l PMSF, pH 7.4) and treated with ultrasonication. Inclusion bodies were collected by centrifugation and were rinsed sequentially with the urea solutions (1, 2, 3, 4, 5, and 6 mol/l) supplemented with 0.1% Triton X-100 to remove unwanted proteins. Purified inclusion bodies were dissolved into the denatured buffer (8 mol/l urea, 50 mmol/l Tris-HCl, 1 mmol/l EDTA, 1 mmol/l PMSF, 2 mol/l DTT). Renaturation was performed by dialyzing sequentially against the denatured buffers with different urea concentrations (6, 5, 4, 3, 2, 1, and 0 mol/l). Protein concentrations were determined by measuring the optical density of the protein solutions stained with Coomassie Brilliant Blue G250 at the wavelength of 595 nm. Polyacrylamide gel electrophoresis (PAGE) was used in characterization of proteins, SDS-PAGE for determination of the molecular weight of protein subunits and Native-PAGE for analysis of the intact oligomeric form of the indicated proteins.

### Extraction and electrophoresis of lipopolysaccharides (LPS)

LPS was extracted from the indicated *P. aeruginosa* strains according to the method described previously [38]. Briefly, 100-ml bacterial cultures were incubated to an OD_600_ of 0.6. Bacterial cells were harvested by centrifugation and resuspended with 5 ml H_2_O. Then cell suspensions were mixed with equal volumes of phenol-water solution for LPS extraction. After incubation at 120 rpm at 68 °C for 30 min, the supernatant containing LPS was collected by centrifugation. Repeat the extraction steps two more times and combine the supernatants. Phenol residue was removed from the LPS solution by dialyzing against pure water for 20 h. Then ethanol was added to the solution at a volume ratio of 2.5:1. The mixture was kept on ice for 2 h followed by centrifugation at 10000×*g* for 15 min for LPS precipitation. LPS was air-dried at 37 °C for 30 min before weighing. SDS-PAGE (14%) was used for separation of LPS and Tricine-SDS-PAGE (10%) was used for analysis of core oligosaccharide. Silver staining was used for visualization of LPS profiles [39].

### Blocking phages with LPS

Intact LPS and core oligosaccharide were prepared from the wild-type strain PAK and the mutant strain SK75 (*wzy* ^-^), respectively. A 1:2 serial dilutions of the LPS preparations were made for the blocking assay. Equal volumes (250 μl) of the LPS dilutions and the phage solutions with the indicated titers were mixed together and incubated statically for 30 min at room temperature. Free phage titers were determined by the double-layer plate method with the logarithmic PAK or SK75 cells as indicators, respectively. Total phage titers were determined in the controls without LPS. The experiments were independently replicated three times. One-way ANOVA was used to analyze the difference between the means of the indicated groups with different treatments.

### Binding efficiency assay

Intact LPS (42.7 mg/ml) and core oligosaccharide (36.0 mg/ml) were used in each assay. Equal volumes (200 μl) of LPS preparations and the purified proteins with the indicated concentrations were mixed together and incubated statically for 15 min. Then 100 μl of the mixture was sampled and added to 200 μl of the indicator phage K8 and K8-T239A with the indicated titers. After incubation at 37 °C for 15 min, free phage titer was determined by the double-layer plate method using the logarithmic PAK or SK75 cells as indicators, respectively. Total phage titers were determined in the controls without LPS. The experiments were independently replicated three times. One-way ANOVA was used to analyze the difference between the means of the indicated groups with different treatments.

### Mass spectrometry analysis of proteome

Viral structural proteins were analyzed according to the method described previously [40]. Briefly, phage particles were purified with Amicon Ultra-0.5 filters (Millipore) and subjected to SDS-PAGE for separation of the structural proteins. The separated proteins were digested with trypsin and analyzed with the LC-MS system Thermo Scientific™ Orbitrap Fusion™. The public protein databases were searched for protein identification by using the online software Mascot (http://www.matrixscience.com/server.html). The identified peptides were aligned with the proteome of phage K8 with the online software MaxQuant (http://www.maxquant.org). The significance threshold was set at *P* ≤ 0.01.

### SEC–MALS analysis

Size exclusion chromatography with multiangle light scattering (SEC-MALS) was used to determine the molar mass of the purified proteins with the Wyatt SEC-MALS system which is equipped with a WTC-015S5 column (7.8 × 300 mm, 5 μm), a DAWN HELEOS-II MALS detector, and an Optilab T-rEX differential refractive index detector (Wyatt Technology, USA). The analyses were performed according to the manufacturer’s protocol. In brief, One-hundred microliter of each protein solutions (0.15–4.05 mg/ml) were loaded for analysis. Bovine serum albumin (BSA) was used as control. The loaded samples were eluted with the buffer (100 mM NaCl, 50 mM Tris-HCl, 1 mM EDTA, 0.02% NaN_3_, pH 7.4) at a flow rate of 0.4 ml/min. The output data was analyzed with the software ASTRA VI (https://www.wyatt.com/products/software/astra.html).

### Abundance of the GP075-like proteins

The distribution of the GP075 homologs in bacteria and phages were investigated with the software PSI-BLAST (Position-Specific Iterated BLAST) with the *E* value threshold lower than 0.001 [41]. The non-redundant protein sequences (nr) database was selected with the data including all non-redundant GenBank CDS translations, PDB, SwissProt, PIR, and PRF, excluding environmental samples from WGS projects. To investigate the distribution of the GP075 homologs across phage groups, the tailed phages (taxid: 28883) and unclassified phages (taxid: 12333) of the nr database was searched.

### Diversity analysis of the gp075 gene

The OSA defective strains SK2, SK15, and SK45 were used as indicators for isolation of the K8 mutants. Single transparent plaques were collected from the bacterial lawns, resuspended in LB medium, and purified with the double-layer plate method. The process was repeated three times. The isolated K8 mutants were validated by spotting 10^5^ phage particles on the double-layer plates with the indicated bacterial lawns. Genomic DNA was extracted from the isolated K8 mutants and used as template for amplification of the *gp075* gene with the primers K8_075-F and K8_075-R listed in Supplementary Table S2. The PCR products were sequenced and aligned for identification of mutations in the *gp075* gene.

To further analyze the genetic variations within the coding region of the *gp075* gene, a pool of single phage plaques was collected from the bacterial lawns of the OSA defective strains SK2, SK15, and SK45. Metagenomic DNA from the mixed plaques was extracted and used as templates for amplification. Three pairs of primers, gp075-F1 and gp075-R1, gp075-F2 and gp075-R2, and gp075-F3 and gp075-R3, were used to amplify three overlapped fragments, 264 bp, 202 bp, and 266 bp, covering the entire coding region of the *gp075* gene (Supplementary Table S2). The PCR products were analyzed with the Illumina Miseq system with max recommended read length up to 2 × 250 bp. Totally 77503, 75418, and 92190 reads as clean data were obtained for each amplified fragment, respectively, and they were assembled into about 38751, 37709, and 46095 sequences representing the coding regions of the *gp075* gene. Amino acid sequences were deduced and alignments were performed to identify mutations.

### Repetitive sequence analysis

The software RepeatAround 2.1 was used to analyze the repetitive DNA sequences of the genome of phage K8 (http://portugene.com/repeataround.html). The length of repetitive DNA sequences range from 3 bp to 64 bp, including direct repeats, inverted repeats, mirror repeats, and complementary repeats [42].

### Protein interactions assay

The BacterioMatch two-hybrid system was used for screening interactions between the viral structural proteins. RS is an *Escherichia coli* strain carrying the reporter gene β-lactamase (*amp*). RS strains carrying recombinant plasmids with bait target protein genes, respectively, were grown in LB medium. One microliter of the cultures (OD_600_ = 0.8) was spot on L-agar plates containing carbenicillin at different concentrations. After incubation at 37 °C for 20 h, bacterial growth on the L-agar plates was evaluated. Negative controls include two groups of strains, one group carrying the plasmid pBT and the target protein genes and the other carrying the plasmid pTRG and the bait protein genes.

### Structure analysis of the GP075-like proteins

The bioinformatic tool BLASTP (https://blast.ncbi.nlm.nih.gov/Blast.cgi) was used for the sequence similarity analysis of the GP075-like proteins, and the online software HHpred (https://toolkit.tuebingen.mpg.de/tools/hhpred) was used for the remote protein homology detection of the GP075 proteins (28).

### Stability analysis

Thermal stability of phage K8 and its derivatives was evaluated by incubating equal phage particles (1 × 10^10^ pfu/ml) at different temperatures (30 °C, 40 °C, 50 °C, 60 °C, 70 °C) for 1 h. The phage samples stored at 4 °C were used as controls used for calculations of survival percentage. The experiment was independently replicated three times. Differences among the mean survival percentages of the groups were analyzed with one-way ANOVA followed by a Tamhane T2 test.

Long-term stability of the K8 phages were analyzed in different buffers, including 0.01 mol/l phosphate buffered saline (pH 7.0 and pH 9.0) and 0.01 mol/l Tris-HCl buffer (pH 5.8). The phage lysates were diluted 300-fold in the indicated buffers. After incubation of the indicated phage solutions at 4 °C or 30 °C for 45 days, phage titers were measured by the double-layer plating method with the wild-type strain PAK and the OSA-defective strain SK75 as the indicators, respectively. Survival percentages of the indicated phages were calculated by comparing the phage titers at the 45th day to that at the 0th day plated on the same indicated bacterial lawns. The experiments were independently replicated three times. One-way ANOVA was used to compare the means of the phage titers tested at day 0 and day 45, respectively. Differences among the mean survival percentages of the groups were analyzed with one-way ANOVA followed by a Tamhane T2 test.

## Supplementary Information

**Additional file 1: Supplementary Table S1.** Bacteria, plasmids, and phages used in this study. **Supplementary Table S2.** Primers used in this study. **Supplementary Table S3.** Molar mass of GP075 and its derivatives. **Supplementary Table S4.** Possible protein interactions identified by the bacterial two-hybrid system ^a^. **Supplementary Table S5.** Mutations of the *gp075* gene of the 41 isolated K8 mutants ^a^. **Supplementary Figure S1.** Protein structure modelling by using the online server SWISS-MODEL. The amino acid sequences of the wild-type protein GP075 and its two variants GP075m and GP075-D7 were uploaded for structural template searching and model building. The upper panels showed the brief descriptions of the secondary structure parameters of each protein. The bottom panels displayed the 3D structure images of the indicated proteins. **Supplementary Figure S2.** SDS-PAGE of the purified viral proteins. GP076: 670 amino acids. GP075: 243 amino acids. GP075m: GP075 with one amino acid substitution T239A. GP075-D7: GP075 with one copy of the 7-aa duplication (APWYSVG). GP075-D14: GP075 with two identical copies of the 7-aa duplication. GP075-D21: GP075 with three identical copies of the 7-aa duplication. M: Premixed Protein Marker (Low) from Takara. **Supplementary Figure S3.** Native-PAGE of the purified viral proteins. GP075: 243 amino acids. GP075m: GP075 with one amino acid substitution T239A. The protein samples for electrophoresis were treated before loading as follows: 1 and 5, the protein samples were mixed with 2×loading buffer (without SDS); 2 and 6, the protein samples were mixed with 2×loading buffer (without SDS) and incubated at 100°C for 10 min. 3 and 7: the protein samples were mixed with 2×loading buffer (containing 4% SDS). 4 and 8: the protein samples were mixed with 2×loading buffer (containing 4% SDS) and incubated at 100°C for 10 min. M: Premixed Protein Marker (Low) from Takara. **Supplementary Figure S4.** Thermostability of the K8 mutants. a. Amino acids sequences alignments of the GP075-like proteins from the isolated K8 mutants. Red letters represent the amino acids duplication or the substitutions. Numbers represent the positions of amino acid residuals in the GP075 protein. b. Thermostability of the K8 mutants. Phages were treated at various temperatures for 1 h before determination of the live phages. The phage titers obtained at the treatment at 4°C were used as controls to calculate the survival percentages. The experiments were independently replicated three times. One-way ANOVA followed by a Tamhane T2 test was performed to compare the means of the groups treated at 70°C. **Supplementary Dataset 1.** LC-MS data. **Supplementary Dataset 2.** Ampliconsequencing data. **Supplementary Dataset 3.**Homologs of GP075.

## Data Availability

Not applicable.

## Abbreviations

LPS: Lipopolysaccharides
OSA: O-specific antigen
WTA: Wall teichoic acid
PCR: Polymerase chain reaction
SDS: Sodium dodecyl sulfate
OTU: Operational taxonomic units
LB: Luria-Bertani
