# Supplementary material for: Variants of a putative baseplate wedge protein extend the host range of Pseudomonas phage K8
Source: Microbiome. 2023 Jan 31;11:18. doi: 10.1186/s40168-022-01459-w (PMC9887876; doi:10.1186/s40168-022-01459-w)
Supplement: Supplementary file 2 — Additional file 1: Supplementary Table S1. Bacteria, plasmids, and phages used in this study. Supplementary Table S2. Primers used in this study. Supplementary Table S3. Molar mass of GP075 and its derivatives. Supplementary Table S4. Possible protein interactions identified by the bacterial two-hybrid system a. Supplementary Table S5. Mutations of the gp075 gene of the 41 isolated K8 mutants a. Supplementary Figure S1. Protein structure modelling by using the online server SWISS-MODEL. The amino acid sequences of the wild-type protein GP075 and its two variants GP075m and GP075-D7 were uploaded for structural template searching and model building. The upper panels showed the brief descriptions of the secondary structure parameters of each protein. The bottom panels displayed the 3D structure images of the indicated proteins. Supplementary Figure S2. SDS-PAGE of the purified viral proteins. GP076: 670 amino acids. GP075: 243 amino acids. GP075m: GP075 with one amino acid substitution T239A. GP075-D7: GP075 with one copy of the 7-aa duplication (APWYSVG). GP075-D14: GP075 with two identical copies of the 7-aa duplication. GP075-D21: GP075 with three identical copies of the 7-aa duplication. M: Premixed Protein Marker (Low) from Takara. Supplementary Figure S3. Native-PAGE of the purified viral proteins. GP075: 243 amino acids. GP075m: GP075 with one amino acid substitution T239A. The protein samples for electrophoresis were treated before loading as follows: 1 and 5, the protein samples were mixed with 2×loading buffer (without SDS); 2 and 6, the protein samples were mixed with 2×loading buffer (without SDS) and incubated at 100°C for 10 min. 3 and 7: the protein samples were mixed with 2×loading buffer (containing 4% SDS). 4 and 8: the protein samples were mixed with 2×loading buffer (containing 4% SDS) and incubated at 100°C for 10 min. M: Premixed Protein Marker (Low) from Takara. Supplementary Figure S4. Thermostability of the K8 mutants. a. Amino acids sequences [file 40168_2022_1459_MOESM1_ESM.zip › Supplementary Information.docx]

**Variants of a putative baseplate wedge protein extend the host range of *Pseudomonas* phage K8**

**Sun et al**

**Supplementary Table 1. Bacteria, plasmids, and phages used in this study**

| **Strain/ Plasmid /Phage** | **Description** | **Source (Reference)** |
| --- | --- | --- |
| ***P. aeruginosa*** |  |  |
| PAK | Wild type *Pseudomonas aeruginosa* strain | ([1](#_ENREF_1)) |
| SK98 | PAK with Tn*5G* transposon inserted at *ssg*, Gm^r^ | ([2](#_ENREF_2)) |
| P2-25 | PAK with Tn*5G* transposon inserted at *wapH*, Gm^r^ | ([3](#_ENREF_3)) |
| M21 | PAK with Tn*5G* transposon inserted at *galU*, Gm^r^ | ([4](#_ENREF_4)) |
| SK2 | PAK with Tn*5G* transposon inserted at *wbpV*, Gm^r^ | ([2](#_ENREF_2)) |
| SK15 | PAK with Tn*5G* transposon inserted at *wbpO*, Gm^r^ | ([2](#_ENREF_2)) |
| SK45 | PAK with Tn*5G* transposon inserted at *wbpR*, Gm^r^ | ([2](#_ENREF_2)) |
| SK75 | PAK with Tn*5G* transposon inserted at *wzy*, Gm^r^ | ([2](#_ENREF_2)) |
| ***E. coli*** |  |  |
| DH5α | *hsdR recA lacZYAF80 lacZDM15* | BRL |
| M15 | Overexpression of *lacI*, Km^r^ | Qiagen |
| RS | BacterioMatch two-hybrid system reporter strain, Km^r^ | Stratagene |
| **Plasmid** |  |  |
| pGEM-T Easy | For cloning PCR products, Ap^r^ | Promega |
| pQE30 | Fusion vector for N-terminal His tag, Ap^r^ | Qiagen |
| pBT | Bait vector encoding phage cI protein, Chl^r^ | Stratagene |
| pTRG | Target vector encoding RNAP α-subunit protein, Tc^r^ | Stratagene |
| pSL1709 | *his-gp075* fusion in pQE30 vector, Ap^r^ | This study |
| pSL1710 | *his-gp075m* fusion in pQE30 vector, Ap^r^ | This study |
| pSL1711 | *his-gp076* fusion in pQE30 vector, Ap^r^ | This study |
| pSL1714 | *his-gp075-D7* fusion in pQE30 vector, Ap^r^ | This study |
| pSL1716 | *his-gp075-D21* fusion in pQE30 vector, Ap^r^ | This study |
| pBT-072 | *gp072* gene cloned in pBT, Chl^r^ | This study |
| pBT-074 | *gp074* gene cloned in pBT, Chl^r^ | This study |
| pBT-075 | *gp075* gene cloned in pBT, Chl^r^ | This study |
| pBT-075m | *gp075m* gene cloned in pBT, Chl^r^ | This study |
| pBT-D7 | *gp075-D7* gene cloned in pBT, Chl^r^ | This study |
| pBT-076 | *gp076* gene cloned in pBT, Chl^r^ | This study |
| pBT-078 | *gp078* gene cloned in pBT, Chl^r^ | This study |
| pTRG-072 | *gp072* gene cloned in pTRG, Tc^r^ | This study |
| pTRG-074 | *gp074* gene cloned in pTRG, Tc^r^ | This study |
| pTRG-075 | *gp075* gene cloned in pTRG, Tc^r^ | This study |
| pTRG-075m | *gp075m* gene cloned in pTRG, Tc^r^ | This study |
| pTRG-D7 | *gp075-D7* gene cloned in pTRG, Tc^r^ | This study |
| pTRG-076 | *gp076* gene cloned in pTRG, Tc^r^ | This study |
| pTRG-078 | *gp078* gene cloned in pTRG, Tc^r^ | This study |
| **Phage** |  |  |
| K5 | *Pseudomonas* phage | ([3](#_ENREF_3)) |
| K8 | *Pseudomonas* phage | ([2](#_ENREF_2)) |
| C11 | *Pseudomonas* phage | ([5](#_ENREF_5)) |
| K8-D7 | A derivative of phage K8 with a 7aa in-frame identical duplication in the protein GP075 | This study |
| K8-E126K | A derivative of phage K8 with a substitution E126K in the protein GP075 | This study |
| K8-S142L | A derivative of phage K8 with a substitution S142L in the protein GP075 | This study |
| K8-L189R | A derivative of phage K8 with a substitution L189R in the protein GP075 | This study |
| K8-P197L | A derivative of phage K8 with a substitution P197L in the protein GP075 | This study |
| K8-T239A | A derivative of phage K8 with a substitution T239A in the protein GP075 | This study |
| K8-X | A derivative of phage K8 with no mutations found in the protein GP075 | This study |

The mutated GP075 proteins were assigned names as follows: GP075m represents the protein with an amino acid substitution T239A. GP075-D7 represents the protein with a 7-aa (APWYSVG) identical duplication between the 113^th^ and 114^th^ amino acid residual. GP075-D21 represents the protein with three 7-aa (APWYSVG) identical duplications between the 113^th^ and 114^th^ amino acid residual. GP072 ang GP074 are predicted as putative baseplate proteins. GP076 and GP078 are predicted as tail fiber proteins.

**Supplementary Table 2. Primers used in this study**

| Primers | Sequence (5'-3') | Target genes |
| --- | --- | --- |
| K8_057-F | AGGGTCGTTCCAGTAAAT | Amplification of the gene *gp057.* |
| K8_057-R | GTCCCCTACAATCTCTCG |  |
| K8-074-F | TCACAGTCAGAACCACAG | Amplification of the gene *gp074.* |
| K8-074-R | TTCTCTGTCAAATTGGTT |  |
| pQE-75-F | CGCGGATCC ATGGCTGTCAACCAATTTGA | The upstream primer for overexpression of the genes *gp075*, *gp075m*, *gp075-7*, and *gp075*-*D7* in pQE30. |
| pQE-75-R | CGGGGTACCTTATGTCACTAGGTTGGTCA | The downstream primer for overexpression of the genes *gp075*, *gp075-7*, and *gp075*-*21* in pQE30. |
| pQE-75m-R | CGGGGTACCTTATGTCACTAGGTTGGCCA | The downstream primer for overexpression of the *gp075m* gene in pQE30. |
| K8_076-F | GAGGACAAGGCGTTGGAGAC | Amplification of the genes *gp076* and *gp077.* |
| K8_077-R | CCCAACTTTTACAGCACCCG |  |
| pQE-76-F | CGCGGATCC ATGGCTGATTACAGTCAACT | Overexpression of the gene *gp076* in pQE30 |
| pQE-76-R | CCCAAGCTTTTAAGAAATGCGCTGCCAG |  |
| gp075-F1 | GTGTAATGATAGAAGGAGAATTCC | Amplification of the 264 bp fragment of 5’ end of the gene *gp075* for amplicon sequencing. |
| gp075-R1 | CACGCTCCGTTCCAG |  |
| gp075-F2 | CAAGATATTTTCAACTATTTTGGATTCG | Amplification of the 202 bp fragment of the central region of the gene *gp075* for amplicon sequencing. |
| gp075-R2 | CTCTAGGAATACTTCAGGAACC |  |
| gp075-F3 | CGAAGCTTATAAATTTGTATTCGG | Amplification of the 266 bp fragment of 3’ end of the gene *gp075* for amplicon sequencing. |
| gp075-R3 | TGTAATCAGCCATTTATAACTCC |  |
| K8_075-F | ATATCACCGTAACTACGGT | Amplification of the gene *gp075* for cloning on the vector pGEM-T Easy. |
| K8_075-R | GTTGACTGTAATCAGCCATT |  |
| K8_078-F | GCTTGGCGTAAGTATCGT | Amplification of the gene *gp078.* |
| K8_078-R | GCCAGAAAGTAGAAAGCC |  |
| 072-pBT-F | CCGGAATTCCATGAGGAGAACAGGCCTACAAG | Amplification of the gene *gp072* for cloning in pBT |
| 072-pBT-R | CCGCTCGAGTTAAGCTTGTGGGACAGCAGTAACA |  |
| 076-pBT-F | CCGGAATTCCATGGCTGATTACAGTCAACTACCTA | Amplification of the gene *gp076* for cloning in pBT |
| 076-pBT-R | CCGCTCGAGCCCTCTATTAAGAAATGCGCT |  |
| 078-pBT-F | CCGGAATTCCATGCCAAACATTATGAAGCCTACGG | Amplification of the gene *gp078* for cloning in pBT |
| 078-pBT-R | CCGCTCGAGTTACAGGGTGGAAGTGATAGCAAAC |  |
| 074-pBT-F | CCGGAATTCCATGGCTGGAATTACAGCAGAA | Amplification of the gene *gp074* for cloning in pBT |
| 074-pBT-R | CCGCTCGAGGTAGTTACGGTGATATCTGCTGC |  |
| 075-pBT-F | ATAAGAATGCGGCCGCAATGGCTGTCAACCAATTTGAC | Upstream primer for amplification of *gp075*, *gp075m*, or *gp075-D7* for cloning in pBT |
| 075-pBT-R | CCGCTCGAGTTATGTCACTAGGTTGGTCAGAATT | Downstream primer for amplification of the gene *gp075* or *gp075-D7* for cloning in pBT |
| 075m-pBT-R | CCGCTCGAGTTATGTCACTAGGTTGGCCAGAATT | Downstream primer for amplification of the gene *gp075m* for cloning in pBT |
| 072-pTRG-F ^c^ | CCGGAATTCAGATGAGGAGAACAGGCCTACAAG | Upstream primer for amplification of *gp072* for cloning in pTRG |
| 076-pTRG-F | CCGGAATTCAGATGGCTGATTACAGTCAACTACCTA | Upstream primer for amplification of *gp076* for cloning in pTRG |
| 078-pTRG-F | CCGGAATTCAGATGCCAAACATTATGAAGCCTACG | Upstream primer for amplification of *gp078* for cloning in pTRG |
| 074-pTRG-F | CCGGAATTCAGATGGCTGGAATTACAGCAGAA | Upstream primer for amplification of *gp074* for cloning in pTRG |
| 075-pTRG-F | ATAAGAATGCGGCCGCAATGGCTGTCAACCAATTTGAC | Upstream primer for amplification of *gp075*, *gp075m*, or *gp075-D7* for cloning in pTRG |

1. The underlined sequences stand for the recognition sites of the restriction enzymes.
2. The primers pQE-75-F and pQE-75-R are used for overexpression of *gp075*, *gp075-D7*, and *gp075-D21* in pQE30, respectively. The primers pQE-75-F and pQE-75m-R are used for overexpression of *gp075m* in pQE30.
3. The downstream primers, which are used in amplification of the genes *gp072*, *gp076*, *gp078*, *gp074*, *gp075*, *gp075m*, and *gp075*-*D7* for cloning in pBT constructs, are also used in the cloning process of pTRG constructs.

**Supplementary Table 3. Molar mass of GP075 and its derivatives**

| **Proteins** | **Mutation sites ^a^** | **M ^b^ (kDa）** | **Mw ^c^ (kDa)** | **Mn ^d^** | **Polydispersity index ^e^** | **Mass Fraction ^f^ (%)** | **Degree of polymerization ^g^** |
| --- | --- | --- | --- | --- | --- | --- | --- |
| BSA | - | 66.43 | 66.68 (±0.265%) | 66.57 (±0.271%) | 1.002 | 95.5 | 1.0 |
| GP075 | Wild-type protein | 26.68 | 2167 (±0.607%) | 1104 (±0.662%) | 1.963 | 100 | 81.2 |
| GP075m | T239A | 26.65 | 4937 (±1.105%) | 2621 (±1.252%) | 1.883 | 100 | 185.3 |
| GP075-D7 | APWYSVG | 27.44 | 6013 (±1.638%) | 4519 (±1.085%) | 1.330 | 100 | 219.1 |
| GP075-D21 | APWYSVGAPWYSVGAPWYSVG | 28.96 | 20540 (±7.314%) | 5044 (±0.893%) | 4.072 | 100 | 709.3 |

1. GP075m has an amino acid substitution T239A. GP075-D7 has one 7-aa identical duplication between the 113^th^ and 114^th^ amino acid residual. The GP075-D21 protein has three 7-aa identical duplications between the 113^th^ and 114^th^ amino acid residual.
2. Molar mass of monomers.
3. Mw: Weight average molar mass. Mw takes into account the molar mass of a chain in determining contributions to the molar mass average.
4. Number average molar mass, Mn. The number average molar mass is the statistical average molar mass of all the polymer chains in the sample.
5. The polydispersity index represents a measure of the broadness of a molar mass distribution of a polymer as defined by Mw/Mn.
6. BSA displays 3 discontinuous peaks of monomer, dimer, and multimer, respectively, and monomer is 95.5% of the loaded BSA in the analysis. All the GP075m protein and its derivatives display only one continuous peak in the analysis, including diverse polymers with different subunits.
7. Average number of repeating units of the GP075m protein and its derivatives defined by Mw/M.

**Supplementary Table 4. Possible protein interactions identified by the bacterial two-hybrid system ^a^**

| Target proteins | Bait proteins | | | | | | |  | Negative controls **^b^** | |  | RS |
| --- | --- | --- | --- | --- | --- | --- | --- | --- | --- | --- | --- | --- |
|  | GP072 **^b^** | GP074 **^c^** | GP075 | GP075m | GP075-D7 | GP076 | GP078 **^b^** |  | pBT | pTRG |  |  |
| GP072 **^b^** | **++++** | **++++** | **+++** | **+++** | **++** | **+++** | **++++** |  | **++++** | **+** |  | **+** |
| GP074 | **+++** | **+++** | **++++** | **+++** | **++++** | **++++** | **++++** |  | **+** | **+** |  |  |
| GP075 | **++++** | **++++** | **++++** | **+++** | **++** | **++++** | **++++** |  | **+** | **+** |  |  |
| GP075m | **++** | **+** | **+++** | **++** | **++** | **++** | **-** |  | **+** | **+** |  |  |
| GP075-D7 | **+** | **-** | **++** | **+** | **++** | **+** | **++** |  | **-** | **+** |  |  |
| GP076 | **++** | **+** | **+++** | **-** | **-** | **+++** | **++** |  | **-** | **±** |  |  |
| GP078 **^b^** | **+++** | **++++** | **++++** | **++** | **++** | **++++** | **++++** |  | **+++** | **+++** |  |  |

**a**: The BacterioMatch two-hybrid system was used for screening interactions between the structural proteins encoded by phage K8, GK8, and D7. RS is an *Escherichia* *coli* strain carrying the reporter gene β-lactamase (*amp*). RS strains carrying plasmids with bait and target protein genes, respectively, were grown in LB medium. One microliter of the cultures (OD_600_ =0.8) was spot on L-agar plates containing carbenicillin at different concentrations. Each strain was spotted twice. After incubation at 37°C for 20 h, bacterial growth on the L-agar plates with 350 μg/ml carbenicillin was evaluated, ‘++++’ for the best growth and ‘-’ for no growth. Negative controls include two groups of strains, one group carrying the plasmid pBT and the target protein genes and the other carrying the plasmid pTRG and the bait protein genes.

**b**: Negative control strains carrying the gene *gp072* or *gp078* grew at rates of ‘+++’ or ‘++++’, suggesting that the bacterial two-hybrid system may not be suitable to detect possible interactions involved with the protein GP072 or GP078. Growth rates were evaluated in the strains without *gp072* or *gp078* genes (enclosed in the green rectangle).

**c**: Strains with growth rates of ‘+++’ or ‘++++’ are recorded as possible interactions occurred between the indicted proteins, and other strains are recorded no interactions between the indicted proteins. Red color represents possible interactions existed in the protein pairs, including GP075 and GP074, GP075 and GP076, GP075m and GP074, GP075-D7 and GP074, and GP074 and GP076. Blue color represents no protein interactions between GP075m and GP076 or GP075-D7 and GP076, respectively.

**Supplementary Table 5. Mutations of the *gp075* gene of the 41 isolated K8 mutants ^a^**

| **Phages** | **Nucleotide changes** | **Amino acid change** | **Phage numbers** |
| --- | --- | --- | --- |
| K8-D7 ^b^ | 339_359dupCGGTGCTCCATGGTACTCGGT | 113_119dupAPWYSVG | 5 |
| K8-E126K ^c^ | 376G>A | E126K | 7 |
| K8-S142L | 425C>T | S142L | 5 |
| K8-L189R | 566T>G | L189R | 6 |
| K8-P197L | 590C>T | P197L | 7 |
| K8-X ^d^ | - | - | 11 |
| Total |  |  | 41 |

1. The isolated K8 mutants have the same host range as K8-T239A
2. A 21-bp in-frame duplication inserted between the 339^th^ and 340^th^ nucleotide or a 7-aa duplication inserted between the 113^th^ and 114^th^ amino acids of the *gp075* gene or the GP075 protein sequence, respectively.
3. Substitutions of nucleotides or amino acid residuals occurred in the indicated positions within the coding region of the *gp075* gene.
4. No mutation was detected within the coding region of the *gp075* gene of the mutant K8-X.

**Supplementary Figure 1**


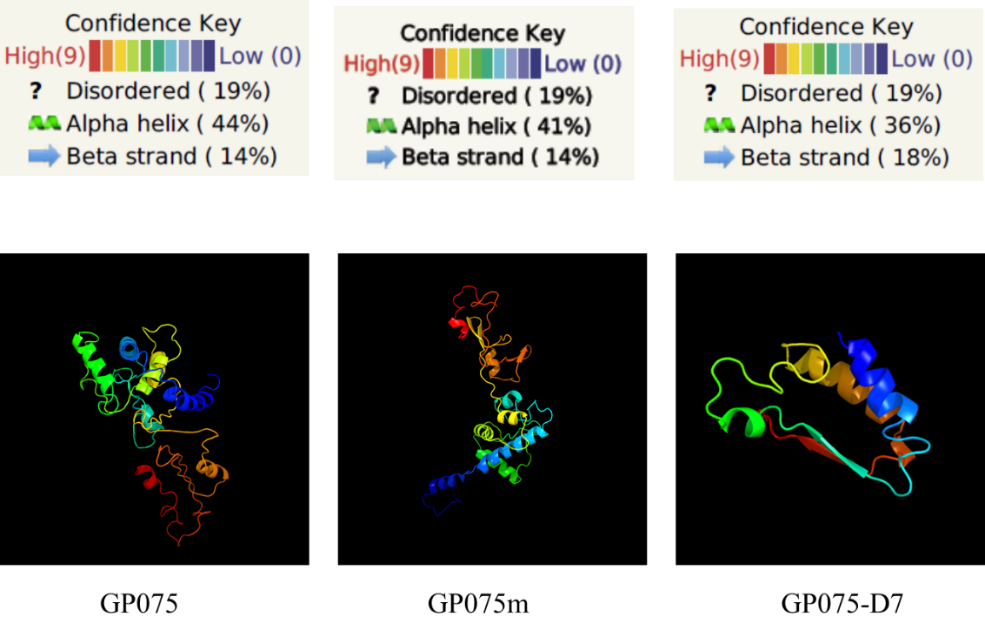


**Supplementary Figure 1**. **Protein structure modelling by using the online server SWISS-MODEL.** The amino acid sequences of the wildtype protein GP075 and its two variants GP075m and GP075-D7 were uploaded for structural template searching and model building. The upper panels showed the brief descriptions of the secondary structure parameters of each protein. The bottom panels displayed the 3D structure images of the indicated proteins.

**Supplementary Figure 2**


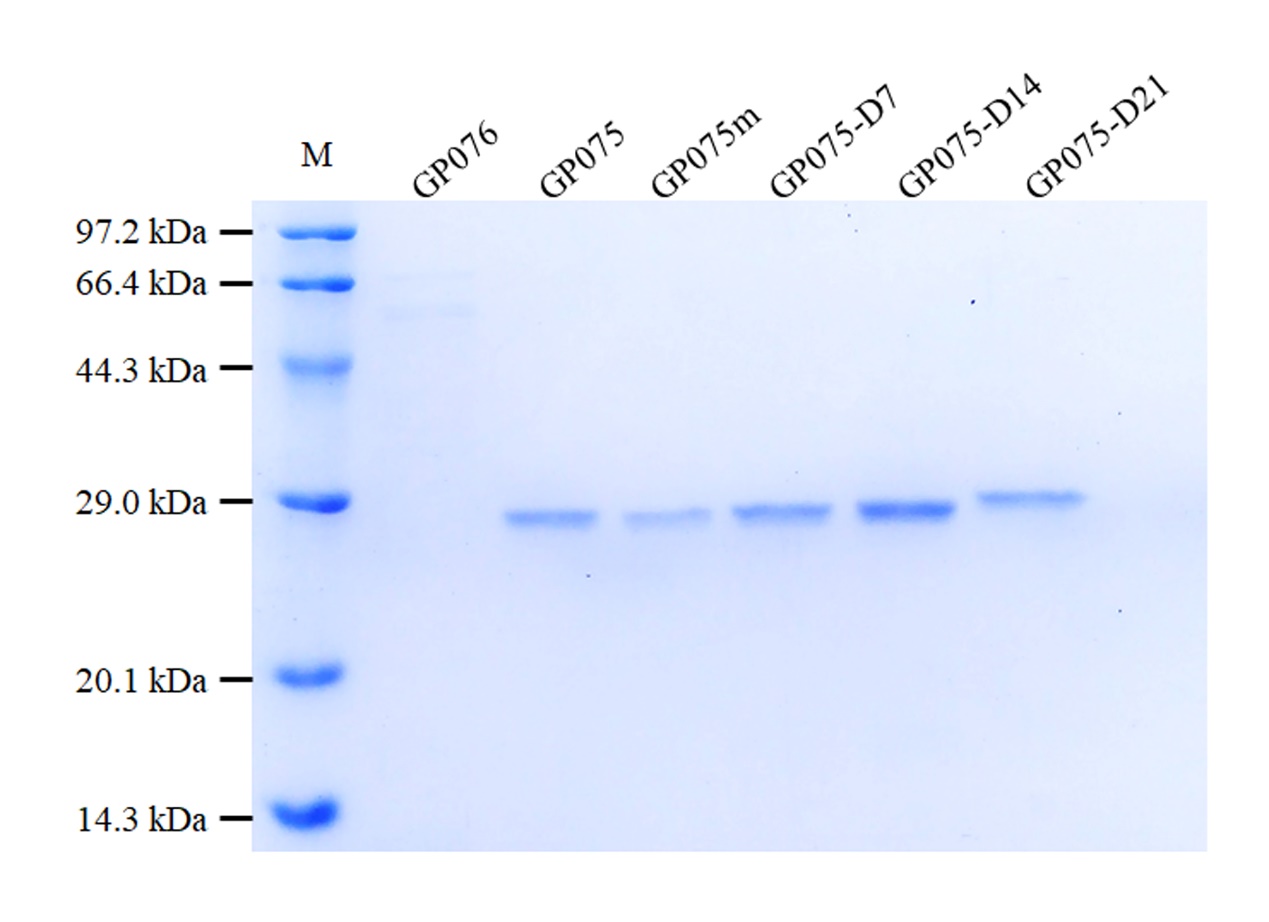


**Supplementary Figure 2. SDS-PAGE of the purified viral proteins.** GP076: 670 amino acids. GP075: 243 amino acids. GP075m: GP075 with one amino acid substitution T239A. GP075-D7: GP075 with one copy of the 7-aa duplication (APWYSVG). GP075-D14: GP075 with two identical copies of the 7-aa duplication. GP075-D21: GP075 with three identical copies of the 7-aa duplication. M: Premixed Protein Marker (Low) from Takara.

**Supplementary Figure 3**


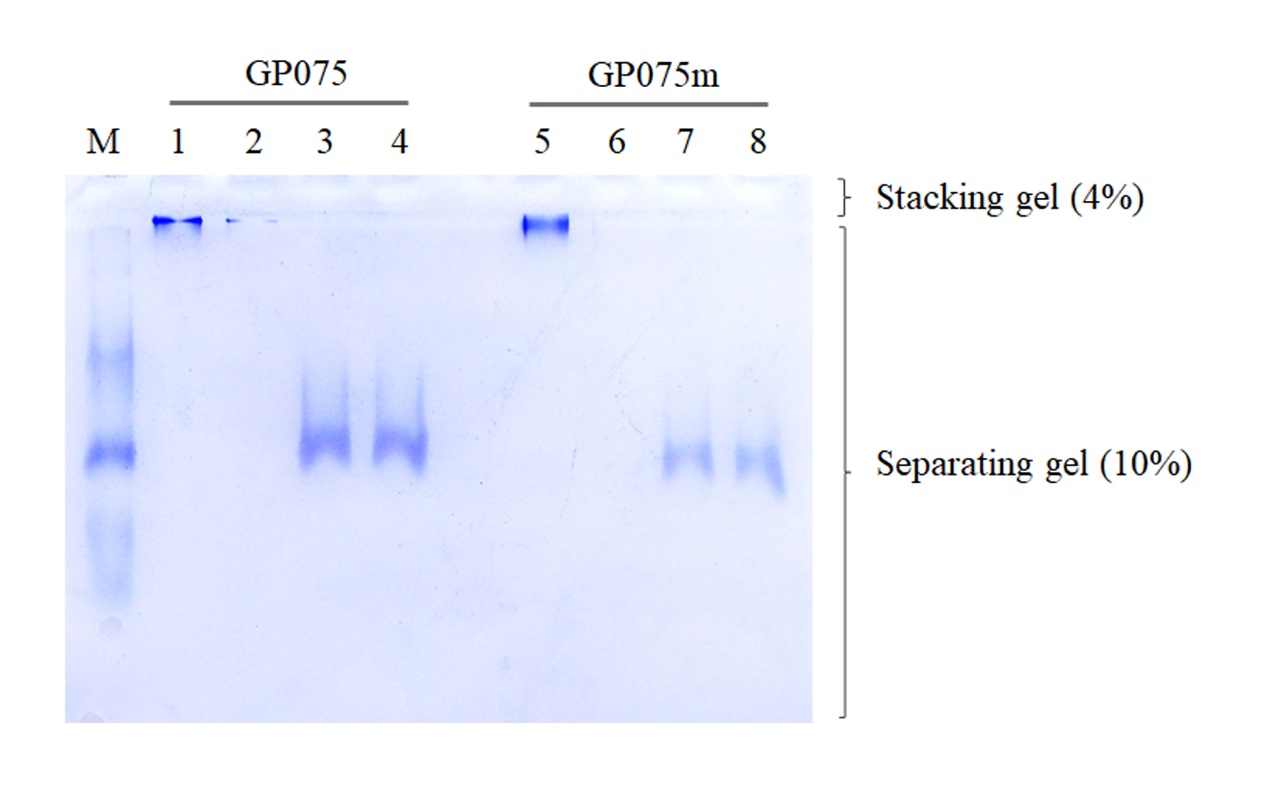


**Supplementary Figure 3. Native-PAGE of the purified viral proteins.** GP075: 243 amino acids. GP075m: GP075 with one amino acid substitution T239A. The protein samples for electrophoresis were treated before loading as follows: 1 and 5, the protein samples were mixed with 2×loading buffer (without SDS); 2 and 6, the protein samples were mixed with 2×loading buffer (without SDS) and incubated at 100°C for 10 min. 3 and 7: the protein samples were mixed with 2×loading buffer (containing 4% SDS). 4 and 8: the protein samples were mixed with 2×loading buffer (containing 4% SDS) and incubated at 100°C for 10 min. M: Premixed Protein Marker (Low) from Takara.

**Supplementary Figure 4**


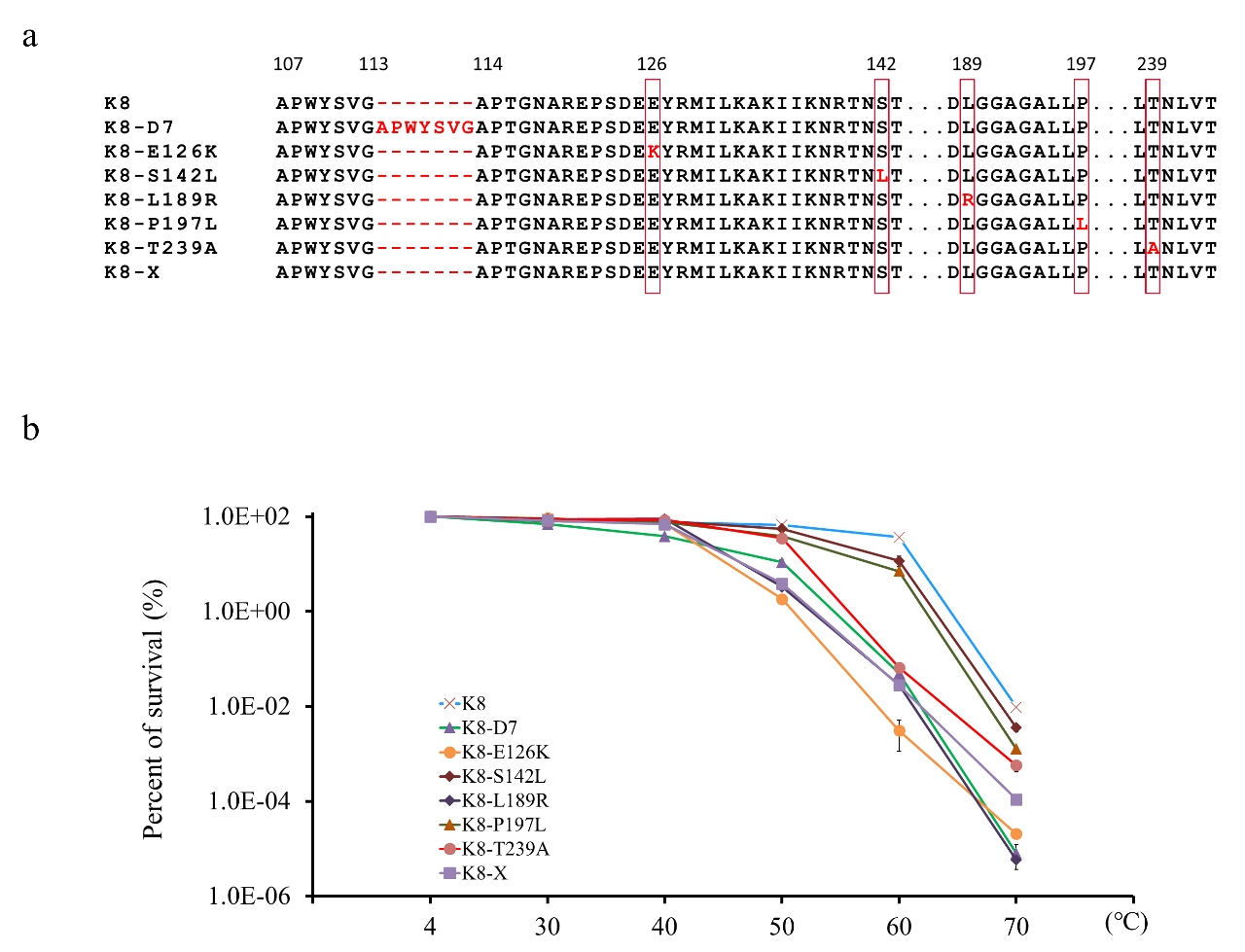


**Supplementary Figure 4. Thermostability of the K8 mutants**. a. Amino acids sequences alignments of the GP075-like proteins from the isolated K8 mutants. Red letters represent the amino acids duplication or the substitutions. Numbers represent the positions of amino acid residuals in the GP075 protein. b. Thermostability of the K8 mutants. Phages were treated at various temperatures for 1 h before determination of the live phages. The phage titers obtained at the treatment at 4°C were used as controls to calculate the survival percentages. The experiments were independently replicated three times. One-way ANOVA followed by a Tamhane T2 test was performed to compare the means of the groups treated at 70°C.

1. Bradley TJ & Khan NH (1974) The production of extracellular lipids by Pseudomonas aeruginosa NCTC 2000 in stationary liquid media containing macrogols. *The Journal of pharmacy and pharmacology* 26(11):900-902.

2. Pan X*, et al.* (2016) Genetic Evidence for O-Specific Antigen as Receptor of Pseudomonas aeruginosa Phage K8 and Its Genomic Analysis. *Frontiers in microbiology* 7:252.

3. Li L*, et al.* (2016) Characterization of Pseudomonas aeruginosa phage K5 genome and identification of its receptor related genes. *Journal of basic microbiology* 56(12):1344-1353.

4. Zhang F*, et al.* (2018) Characterization of a novel lytic podovirus O4 of Pseudomonas aeruginosa. *Archives of virology* 163(9):2377-2383.

5. Cui X*, et al.* (2016) Characterization of Pseudomonas aeruginosa Phage C11 and Identification of Host Genes Required for Virion Maturation. *Scientific reports* 6:39130.
